# A gene expression fingerprint of *C. elegans *embryonic motor neurons

**DOI:** 10.1186/1471-2164-6-42

**Published:** 2005-03-21

**Authors:** Rebecca M Fox, Stephen E Von Stetina, Susan J Barlow, Christian Shaffer, Kellen L Olszewski, Jason H Moore, Denis Dupuy, Marc Vidal, David M Miller

**Affiliations:** 1 Department of Cell and Developmental Biology, Vanderbilt University, Nashville, TN 37232-8240, USA; 2 CHGR, Bioinformatics Core, Vanderbilt University, Nashville, TN 37232-0700, USA; 3 Dartmouth Medical School, Computational Genetics Laboratory, 706 Rubin Building, HB7937, One Medical Center Drive, Lebanon, NH 03756, USA; 4 Center for Cancer Systems Biology and Department of Cancer Biology, Dana-Farber Cancer Institute and Department of Genetics, Harvard Medical School, Boston, Massachusetts 02115, USA

## Abstract

**Background:**

Differential gene expression specifies the highly diverse cell types that constitute the nervous system. With its sequenced genome and simple, well-defined neuroanatomy, the nematode *C. elegans *is a useful model system in which to correlate gene expression with neuron identity. The UNC-4 transcription factor is expressed in thirteen embryonic motor neurons where it specifies axonal morphology and synaptic function. These cells can be marked with an *unc-4*::GFP reporter transgene. Here we describe a powerful strategy, Micro-Array Profiling of *C. elegans *cells (MAPCeL), and confirm that this approach provides a comprehensive gene expression profile of *unc-4*::GFP motor neurons *in vivo*.

**Results:**

Fluorescence Activated Cell Sorting (FACS) was used to isolate *unc-4*::GFP neurons from primary cultures of *C. elegans *embryonic cells. Microarray experiments detected 6,217 unique transcripts of which ~1,000 are enriched in *unc-4*::GFP neurons relative to the average nematode embryonic cell. The reliability of these data was validated by the detection of known cell-specific transcripts and by expression in UNC-4 motor neurons of GFP reporters derived from the enriched data set. In addition to genes involved in neurotransmitter packaging and release, the microarray data include transcripts for receptors to a remarkably wide variety of signaling molecules. The added presence of a robust array of G-protein pathway components is indicative of complex and highly integrated mechanisms for modulating motor neuron activity. Over half of the enriched genes (537) have human homologs, a finding that could reflect substantial overlap with the gene expression repertoire of mammalian motor neurons.

**Conclusion:**

We have described a microarray-based method, MAPCeL, for profiling gene expression in specific *C. elegans *motor neurons and provide evidence that this approach can reveal candidate genes for key roles in the differentiation and function of these cells. These methods can now be applied to generate a gene expression map of the *C. elegans *nervous system.

## Background

The nervous system is assembled from disparate classes of neurons that together define the overall properties of the network. The specific functions of these neurons are governed by genetic programs that control cell fate [1]. Thus, a key to understanding the molecular basis for neural function is to establish the gene expression blueprint that orchestrates neuronal differentiation. With its simple, well-defined nervous system and powerful genetics, the nematode *C. elegans *is a useful model system for addressing this issue. The *C. elegans *hermaphrodite nervous system is composed of exactly 302 neurons. The morphology and connectivity of each one of these neurons has been defined at high resolution [2]. In addition, the birth of each neuroblast is embedded in a lineage diagram of every cell division in *C. elegans *development [3,4]. The *C. elegans *genome is fully sequenced and contains ~20,000 predicted genes [5]. At a fundamental level, the identity of a given class of neuron is defined by a unique combination of these genes. In principle, microarray-based strategies could be employed to establish these cell-specific patterns of gene expression. However, the small size of the nematode has limited access to individual cells for molecular analysis. Here we describe a strategy, MAPCeL (Micro-Array Profiling of *C. elegans *Cells) that overcomes these obstacles to generate neuron-specific gene expression profiles.

MAPCeL exploits recently developed methods of culturing *C. elegans *embryonic cells. GFP markers for specific classes of neurons and muscle cells are expressed *in vitro *and can be used to identify the corresponding differentiated cell types. We established that these GFP cells arise at a frequency predicted by their abundance in the intact embryo and display normal morphological, molecular, and physiological characteristics [6]. For example, a GFP reporter for the *unc-4 *homeodomain transcription factor gene is expressed in 13 motor neurons out of a total 550 cells in the mature embryo (Figs. 2D, 5A) [7]. *In vitro*, we detected a comparable fraction (~2%) of *unc-4*::GFP cells. Moreover, cultured *unc-4*::GFP cells adopt neuronal-like processes and express molecular markers also seen *in vivo *(Fig. 2D–E) [6]. On the basis of these results, we have profiled cultured *unc-4*::GFP neurons with the expectation that this approach will provide a comprehensive picture of genes expressed in these motor neurons *in vivo*.

**Figure 1:**
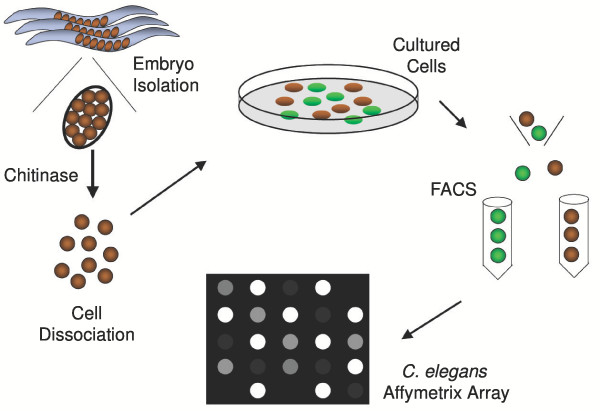
**MAPCeL strategy for profiling *C. elegans *GFP neurons**. Embryos are isolated from gravid adults and treated with chitinase to degrade the egg shell. Embryonic cells are cultured for 24 hours and enriched by FACS. Amplified, labeled aRNA is hybridized to the Affymetrix *C. elegans *array.

**Figure 2:**
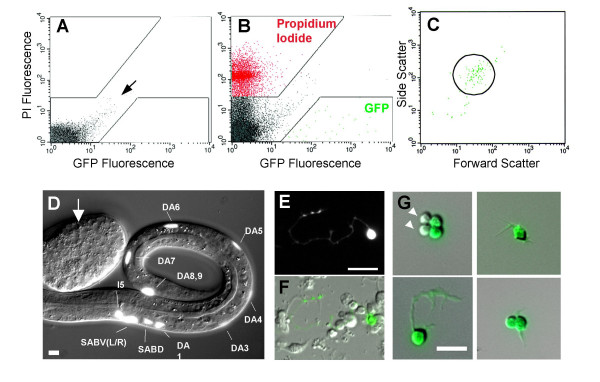
**Isolation of *unc-4::GFP *neurons by FACS**. A. Fluorescence intensity profile of wildtype (non-GFP) cells. Boxed areas exclude autofluorescent7 cells (arrow). B. *unc-4*::GFP cells are gated to exclude propidium iodide-stained (non-viable) cells. C. Light scattering gate for GFP-positive cells (circle) to exclude cell clumps and debris. D. Combined fluorescence and DIC image of *unc-4*::GFP labeled motor neurons in L1 larva. (DA2 is not visible here.) Arrow points to embryo at stage (< 400 min) prior to *unc-4*::GFP expression. E, F. Fluorescence and DIC images of 24 hr culture from *unc-4*::GFP embryos. G. *unc-4*::GFP neurons after enrichment by FACS. Arrow heads point to rare (~10%) non-GFP cells. Scale bars are 5 microns.

**Figure 3:**
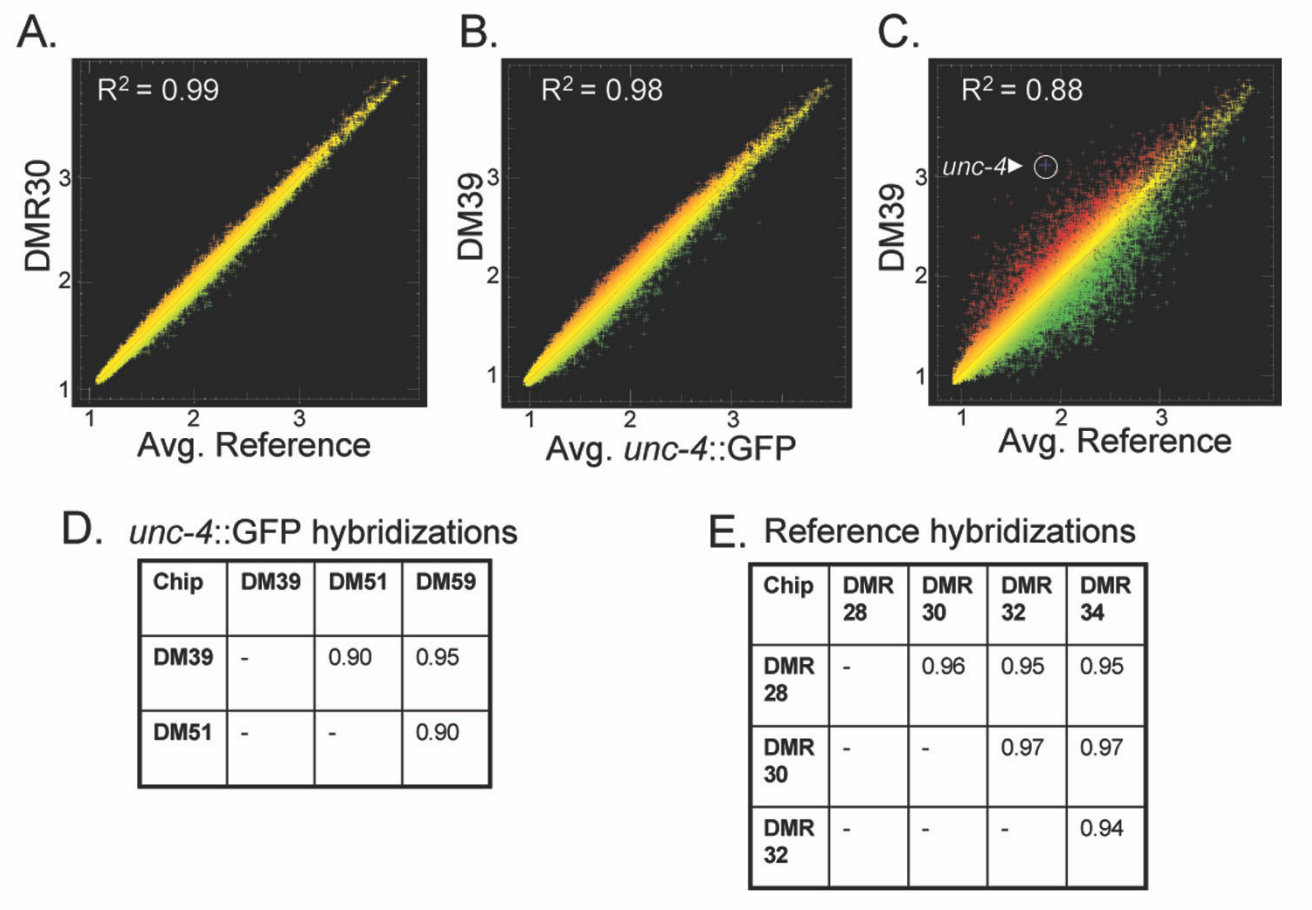
**Coefficients of Determination (R^2^) for individual hybridizations**. A. Scatter plot of normalized intensity values (log base 2) for representative hybridization (DMR30) from all cells (Reference) compared to the average intensity of four Reference hybridizations. B. Scatter plot of representative *unc-4*::GFP hybridization (DM39) compared to the average intensities for all three *unc-4*::GFP hybridizations. C. Results of single *unc-4*::GFP hybridization (DM39) (red) compared to average Reference intensities (green) to identify transcripts showing differential expression. The *unc-4 *transcript (arrowhead) is highly enriched (~13×) in *unc-4*::GFP neurons D. R^2 ^values for all pairwise combinations of *unc-4*::GFP hybridizations. E. R^2^values for all pairwise combinations of Reference (i.e. all cells) hybridizations.

**Figure 4:**
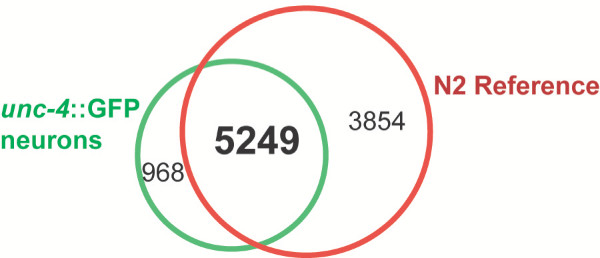
**Comparison of Expressed Genes (EGs) in *unc-4*::GFP neurons vs all cells (N2 reference)**. 968 transcripts are detected exclusively in *unc-4*::GFP motor neurons and 3854 transcripts are detected exclusively in the N2 reference data set. 5249 transcripts are detected in both data sets.

**Figure 5:**
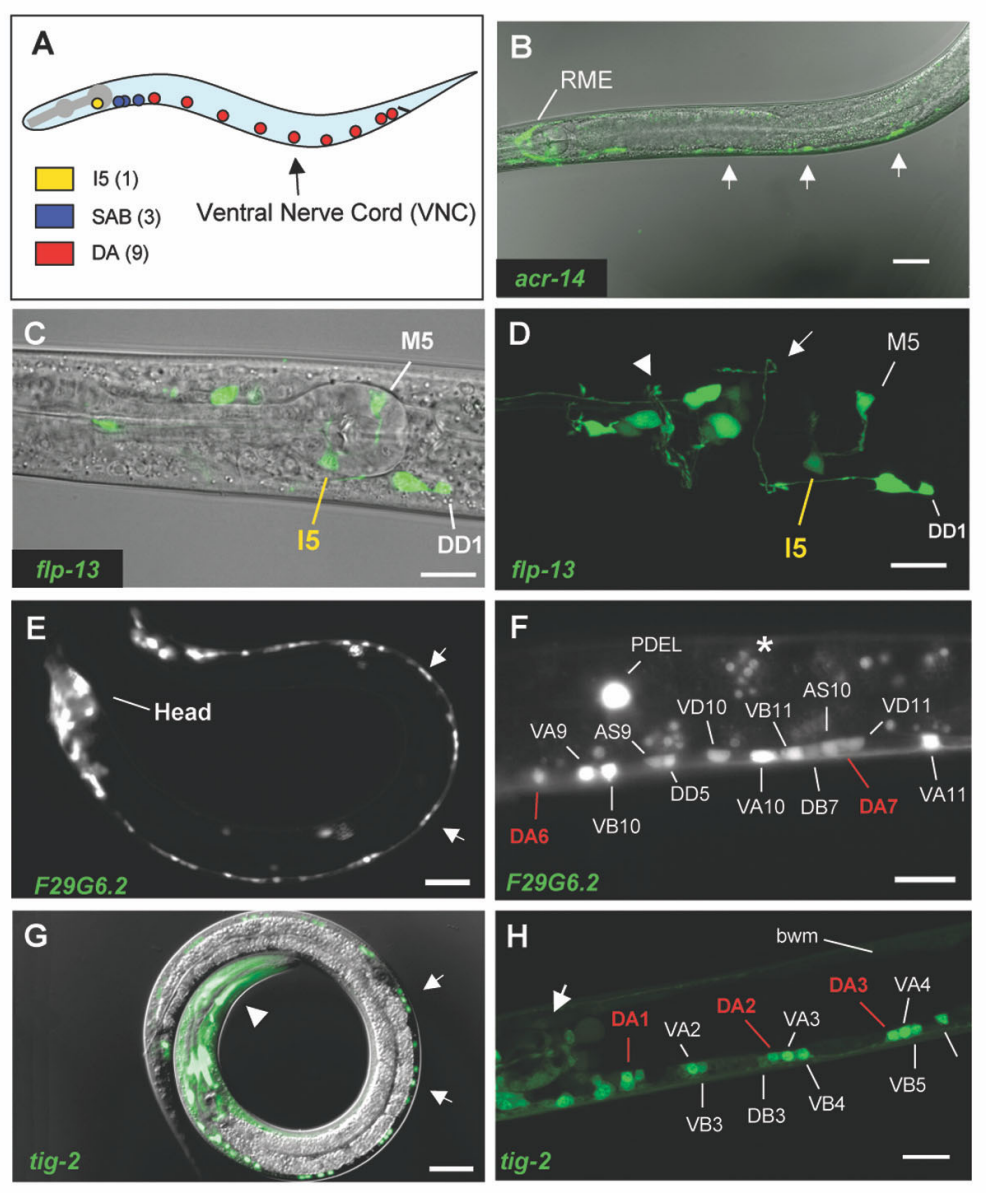
**GFP reporters validate UNC-4 motor neuron genes**. A. *unc-4*::GFP is expressed in 13 embryonically-derived motor neurons. Colored circles indicate approximate location of UNC-4 motor neuron soma in newly hatched L1 larva. Transgenic animals expressing GFP reporters for representative UNC-4 motor neuron genes. Anterior to left. B. *acr-14*. Confocal GFP/DIC projection. *acr-14*::GFP is expressed in RME in the head and in ventral nerve cord (VNC) motor neurons (arrows). C, D. *flp-13*. Confocal DIC/GFP image of head region. (C) and matched confocal GFP projection (D). Note DD1 commissure to dorsal side (white arrow). Arrowhead marks nerve ring. E, F. F29G6.2. Arrows point to VNC motor neurons (L2 Larva) (E). Posterior view of VNC showing F29G6.2::GFP expression in all VNC motor neurons. Asterisk marks gut autofluorescence (F). G, H. *tig-2*. DIC/GFP image of L2 larva. Note GFP expression in VNC motor neurons (arrows) and in head muscles (arrowhead) (G). Confocal projection of anterior VNC. *tig-2*::GFP is detected in A and B class motor neurons, pharyngeal muscle (arrow) and body wall muscle (bwm) (H). Scale bars are 10 microns in panels C, D, F, H and 20 microns in B, E, G.

We describe methods for isolating *unc-4*::GFP-labeled neurons by Fluorescence Activated Cell Sorting (FACS). mRNA from these cells is amplified, labeled and hybridized to the *C. elegans *Affymetrix Gene Chip. A comparison to microarray data derived from all embryonic cells reveals ~1000 genes with significantly higher levels of expression in *unc-4*::GFP neurons. The validity of these data is supported by the inclusion of genes known to be expressed in these neurons *in vivo *and by the generation of new GFP reporters from previously uncharacterized genes on this list. We conclude that MAPCeL offers a reliable strategy for profiling gene expression of a specific motor neuron class. Using this approach, we have provided, for the first time, a comprehensive picture of gene expression in a subset of *C. elegans *motor neurons. We expect that MAPCeL can now be applied to other *C. elegans *neurons and thereby link specific neuronal fates with unique combinations of differentially expressed genes.

## Results

### Profiling strategy

*unc-4*::GFP is expressed in 13 embryonic motor neurons; (1) I5 (pharynx), (3) SAB (retrovesicular ganglion), and (9) DA (ventral nerve cord) (Figs 2D, 5A) [7]. Although each of the motor neuron classes is morphologically distinct, the DA and SAB motor neurons, which constitute the majority (12/13) of *unc-4*:GFP neurons, also share several characteristics including common presynaptic inputs, anteriorly directed axonal processes, cholinergic activity, and similar defects in *unc-4 *mutants [2,8]. It is therefore reasonable to assume that many of the same genes would be expressed in both of these motor neuron classes and that these could be revealed in microarray experiments.

A schematic of our approach to profile *unc-4::*GFP cells is presented in Fig. 1. *C. elegans *embryonic cells were cultured for 24 hr to allow differentiation of GFP-labeled motor neurons. *unc-4*::GFP cells are rarely observed in freshly dissociated preparations but constitute about 2% of all cells after 1 day in culture. The delayed appearance of *unc-4*::GFP cells in culture is consistent with the developmental timing of *unc-4*::GFP expression *in vivo*; *unc-4*::GFP motor neurons are normally generated after morphogenesis is initiated [7]. These older embryos are not dissociated by our methods [6]. Fluorescence Activated Cell Sorting (FACS) is used to isolate enriched (~90%) populations of *unc-4*::GFP cells. RNA is extracted, amplified, and labeled for application to the *C. elegans *Affymetrix Gene Chip.

### *unc-4*::GFP motor neurons are isolated by FACS

It is necessary to plate freshly dissociated embryonic cells on a solid substrate to promote differentiation and to prevent clumping. Although *C. elegans *neurons show extensive morphological differentiation on peanut lectin-coated glass they also adhere avidly and cannot be easily removed. We discovered that cells plated on poly-L-lysine coated surfaces also differentiate but can be readily dissociated from substrate by gentle trituration. A fluorescence profile was established for cells from the non-GFP wildtype strain (N2) to identify autofluorescent intestinal cells. Because these cells autofluoresce in both the Propidium Iodide (PI) and GFP channels, they are largely restricted to the diagonal axis of this scatter plot (Fig 2A). PI was added immediately prior to sorting to stain damaged cells (~20%). Separate experiments with PI-stained wildtype cells and with cells from *unc-4*::GFP embryos were used to establish sorting gates for PI and GFP-labeled cells, respectively (Fig 2B). As shown in Fig 2C, viable *unc-4*::GFP neurons were simultaneously gated by light scattering parameters. This gate was established empirically to achieve ~90% enrichment of *unc-4*::GFP labeled cells (Fig 2G). We typically obtained about 40,000 *unc-4*::GFP neurons from each sort. RNA from the equivalent of 100,000 *unc-4*::GFP neurons was pooled for each separate microarray experiment. We will refer to microarray results from *unc-4*::GFP marked cells as the "*unc-4*::GFP motor neuron" data set. Reference RNA was extracted from all viable cells sorted from a 24 hr culture of wildtype embryonic cells. Microarray results with this "Reference" data set should reflect transcript levels in the average differentiated embryonic cell.

### Microarray experiments yield reproducible profiles

Data obtained from successive hybridizations of two separate arrays with the same labeled probe yielded a coefficient of determination, R^2 ^= 0.99 (data not shown). This result indicates that potential differences between individual Affymetrix arrays or hybridization and scanning procedures are not significant sources of error. The overall concurrence of the experimental (*unc-4*::GFP motor neuron) and Reference data is illustrated graphically in the scatter plots shown in panels A and B of Fig 3. To assess the reproducibility of sample preparation methods (e.g. FACS isolation, RNA extraction, amplification, labeling, etc.), R^2 ^was calculated for each pairwise combination of independent samples. An average R^2 ^of 0.96 (n = 4) was calculated for the wildtype (N2) reference samples (Fig 3E); average R^2 ^was 0.92 (n = 3) for the *unc-4*::GFP motor neuron data set (Fig 3D). These values are indicative of highly similar samples and thereby show that our methods are reliable.

### Detecting Expressed Genes (EGs)

Differential hybridization to perfect match (PM) vs mismatch (MM) oligo probes on the Affymetrix chip was used to identify transcripts reliably detected as "present" in the Reference and *unc-4*::GFP motor neuron data sets (see Methods, Additional Files 4, 5). This list was adjusted in two ways for the *unc-4*::GFP motor neuron data set to arrive at a more accurate representation of Expressed Genes (EGs) (Additional Files 7, 17). In the first instance, transcripts that were statistically downregulated in *unc-4*::GFP motor neurons relative to the wildtype reference were removed from the "present" list as these are likely to be detected because they are actually highly enriched in contaminating the non-GFP cells (~10%) (Additional File 6). Conversely, we included transcripts that were considered enriched according to our statistical methods but originally scored as "absent" on the basis of PM vs MM signals used by Affymetrix MAS 5.0 software (see Methods, Additional file 17). This second adjustment simply acknowledges that enriched transcripts are clearly expressed and therefore should be scored as "present." We refer to the transcripts in these modified lists as EGs (Expressed Genes). A total of 9,103 EGs were detected in the Reference data set and 6,217 EGs in the *unc-4*::GFP motor neuron data set (Fig 4) (Additional Files 4, 7). Overall 10,071 unique transcripts were detected in these experiments or about 50% of all predicted *C. elegans *ORFs [9] (Additional file 8). These results are comparable to microarray data from whole embryos that also detected about half of the predicted *C. elegans *genes [10]. Genes that are not detected may be expressed in a relatively small number of cells. This point is substantiated by our finding that 968 EGs in the *unc-4*::GFP motor neuron data set are not scored as present in the Reference data set (Additional file 15, Fig 4). For example, the transcription factor UNC-3 is normally expressed in a small subset of embryonic neurons including the DAs [11]. The *unc-3 *transcript is enriched in the *unc-4*::GFP motor neuron data set (Table 2, Additional file 9) but is not detected in the Reference (Additional file 4). Thus, it seems likely that the overall number of EGs should increase as additional classes of embryonic cells are profiled (RMF, SEV, SJB, DMM, unpublished data).

**Table 1:** Expression of promoter-GFP reporters for transcripts enriched in *unc-4*::GFP motor neuron data set. Reporters were examined for expression in DA, SAB and I5 neurons. 15/18 reporters showed GFP expression (bold type) in these cells. GFP reporters are listed according to statistical rank. All GFP-positive reporters were visible in embryos (data not shown) but were scored in larval animals to ease neuron identification.

**Rank**	**Cosmid**	**Gene**	**Protein**	**UNC-4 neuron**	**Other cells**
15	F33D4.3	*flp-13*	neuropeptide	**I5**	ASE, ASG, ASK, BAG, DD, M3, M5, head neurons [67]
17	C11D2.6	*nca-1*	Ca^++^channel	**DA**	DB, VA, VB, head/tail neurons
56	F09C3.2		phosphatase	**DA**	VA, VB, VD, DB, intestine, hypodermis
98	T19C4.5		novel		no GFP
161	T23D8.2	*tsp-7*	tetraspanin	**DA**	all VNC motor neurons, head/tail neurons, touch neurons
165	CC4.2	*nlp-15*	neuropeptide		DD, head/tail neurons, body muscles, pharyngeal muscle [105]
210	F29G6.2		novel	**DA**	DB, touch neurons, pharyngeal neurons, head neurons
215	F39G3.8	*tig-2*	TGF-β	**DA**	VA, VB, DB, body wall muscle, touch neurons, pharyngeal muscle
233	F55C12.4		novel	**DA**	VB, DB, DD, AS, VD
234	E03D2.2	*nlp-9*	neuropeptide		VA, intestine, head neurons [105]
239	ZC21.2	*trp-1*	Ca^++^channel	**DA**	DB, VA, VB
254	Y47D3B.2A	*nlp-21*	neuropeptide	**DA**	DB, VA, VB, AS, body muscle, head neurons, intestine [72]
329	F36A2.4	*twk-30*	K^+ ^channel	**DA**	all VNC motor neurons [106]
377	C18H9.7	*rpy-1*	rapsyn	**DA**	VD, AS, VB, DB, body muscles
593	F43C9.4a	*mig-13*	CUB domain	**DA**	DB, ant. VNC motor neurons, pharyngeal/intestinal valves, hypodermis [107]
782	T05C12.2	*acr-14*	nAChR	**DA**	VB, AS, DB, DD, HSN, VC4 & 5, AIY, head neurons, muscles, intestine
788	T27A1.6	*mab-9*	transcrip. factor	**DA**	DD, DB, VD, AS
877	K02E10.8	*syg-1*	Ig Domain	**DA**	VA, HSN and other neurons

**Table 2:** Summary of genes with enriched transcripts in *unc-4::*GFP neurons. Genes are organized into categories according to molecular function (KOG or other description ). Gene families or functional groups with potential functions in neurons are emphasized in this list. Statistical rank is indicated for each transcript.

**Cosmid Name**	**Common Name**	**Rank**	**KOG (Other description)**
***Axon Guidance and Outgrowth***			
			
B0273.4a	*unc-5*	934	Netrin transmembrane receptor unc-5
T19B4.7	*unc-40*	188	Receptor mediating netrin-dependent axon guidance
F41C6.1	*unc-6*	616	Netrin, axonal chemotropic factor
F56D1.4a	*clr-1*	754	Protein tyrosine phosphatase
M79.1a	*abl-1*	882	Protein tyrosine kinase
F09B9.2	*unc-115*	696	Actin-binding LIM Zn-finger protein Limatin involved in axon guidance
B0350.2	*unc-44*	449	Ankyrin
C01G10.11a	*unc-76*	182	Kinesin-associated fasciculation and elongation protein involved in axonal transport
K10D3.2	*unc-14*	763	(RUN domain protein required for axonogenesis and sex myoblast migration)

***Wingless Signaling***			
			
K10B4.6	*cwn-1*	216	Wnt family of developmental regulators
Y71F9B.5a	*lin-17*	899	Smoothened and related G-protein-coupled receptors (Frizzled Receptor)

***Acetylcholine Receptor Subunits***			
			
K11G12.2	*acr-2*	66	Acetylcholine receptor
R01E6.4	*acr-12*	292	Acetylcholine receptor
F21F3.5	*unc-38*	399	Acetylcholine receptor
T05C12.2	*acr-14*	782	Acetylcholine receptor
Y110A7A.3	*unc-63*	800	Acetylcholine receptor
F21A3.7		212	Acetylcholine receptor
Y105E8A.7	*lev-10*	323	Cubilin, multiligand receptor mediating cobalamin absorption
T14A8.1	*ric-3*	237	Unnamed protein (Required for nAChR assembly/trafficking)

***Ligand-gated Ion Channel***			
			
ZC196.7	*glr-5*	792	Glutamate-gated kainate-type ion channel receptor subunit GluR5 and related subunits
T27E9.9		41	Ligand-gated ion channel (glycine/GABA)
Y71D11A.5		502	Ligand-gated ion channel (glycine/GABA)
Y46G5A.30	*snf-5*	686	Sodium-neurotransmitter symporter
C09E8.1a		578	Sodium-neurotransmitter symporter

***G-proteins***			
			
M01D7.7a	*egl-30*	124	G protein subunit Galphaq/Galphay, small G protein superfamily
F08B6.2	*gpc-2*	469	G protein gamma subunit
F56H9.4	*gpa-9*	638	G-protein alpha subunit (small G protein superfamily)

***G-protein Pathway Components***			
			
F28C1.2	*egl-10*	872	G protein signaling regulators
C05B5.7	*rgs-1*	486	G protein signaling regulators
F17C8.1	*acy-1*	884	Adenylyl cyclase
R07E4.6	*kin-2*	467	cAMP-dependent protein kinase types I and II, regulatory subunit
C17F4.6	*gcy-19*	213	Natriuretic peptide receptor, guanylate cyclase
C50H2.2	*egl-47*	432	(Gα_o _coupled receptor)
F57F5.5	*pkc-1*	747	Serine/threonine protein kinase
F39B2.8		928	Predicted membrane protein
C24A8.4		1010	STE20-like serine/threonine kinase MST

***Neuropeptides***			
			
W07E11.3	*flp-2*	31	Unnamed protein (FMRF-like peptide)
C18D1.3	*flp-4*	691	(FMRF-like peptide)
C03G5.7	*flp-5*	562	(FMRF-like peptide)
F33D4.3	*flp-13*	15	Unnamed protein (FMRF-like peptide)
E03D2.2	*nlp-9*	234	Unnamed protein (Neuropeptide-like protein)
CC4.2	*nlp-15*	165	Unnamed protein (Neuropeptide-like protein)
Y47D3B.2a	*nlp-21*	254	Unnamed protein (Neuropeptide-like protein)
F13B12.5	*ins-1*	200	(Insulin-like peptide)
T28B8.2	*ins-18*	767	Unnamed protein (Insulin-like peptide)
F56A11.5		928	Uncharacterized Fe-S protein

***Neuropeptide Processing and Secretion***			
			
T03D8.3		613	Proprotein convertase (PC) 2 chaperone involved in secretion (neuroendocrine protein 7B2)
C32E8.7	*ric-19*	20	Secretory vesicle-associated protein ICA69, contains Arfaptin domain
ZK897.1	*unc-31*	588	Ca2+-dependent activator protein

***Neuropeptide Receptor***			
			
T05A1.1	*npr-2*	362	7 transmembrane receptor (neuropeptide receptor family)
F59D12.1		797	7 transmembrane receptor (rhodopsin-like GPCR)
T07D4.1		77	7 transmembrane receptor (rhodopsin-like GPCR)
Y62E10A.4		275	7-transmembrane receptor (rhodopsin-like GPCR)
K07E8.5		452	Unnamed protein (FMRF receptor)
C35A11.1		814	Unnamed protein (rhodopsin-like GPCR)
ZC84.4		708	7 transmembrane receptor (rhodopsin-like GPCR)
F56B6.5	*uvt-6*	629	7 transmembrane receptor (somatostatin receptor)
F56A11.5		928	Uncharacterized Fe-S protein (Neuropeptide receptor activity)

### Selected *C. elegans *genes are enriched in UNC-4 motor neurons

A majority of transcripts in the Reference and *unc-4*::GFP motor neuron data sets show comparable levels of expression (Fig 2). Many of these transcripts are likely to encode core functions required in every cell. Other transcripts in this group could be limited to subsets of embryonic cells that include UNC-4 motor neurons. Genes that are widely expressed in neurons, for example, may not be detectably enriched in *unc-4*::GFP motor neurons in comparison to the Reference because neurons constitute a significant fraction (~40%) of all cells in the embryo. To illustrate this point, we note that UNC-64 (Syntaxin), an integral component of the neurotransmitter release mechanism and therefore expressed in most neurons [12,13], is detected in the *unc-4*::GFP motor neuron data set but is not enriched (Table 2, Additional Files 7, 14).

As graphically illustrated in the scatter plot shown in Fig. 3C, subsets of genes in the *unc-4*::GFP motor neuron data set are differentially expressed relative to the average expression levels for all cells in the Reference data set (R^2 ^= 0.88). As expected for a gene that is selectively expressed in *unc-4*::GFP neurons, the hybridization signal for the *unc-4 *transcript is highly elevated (13×) in comparison to all cells. Significant numbers of genes are also under-expressed in UNC-4 motor neurons relative to other embryonic cells. Transcripts showing ≥ 1.7× fold intensity difference in the *unc-4*::GFP motor neuron vs Reference data sets were defined using SAM statistics at a False Discovery Rate (FDR) of ≤ 1%. By these criteria 1012 genes are enriched (red) in UNC-4 motor neurons (Additional file 9) whereas 1596 transcripts are depleted (green) (Additional file 10). The threshold of ≥ 1.7× fold was defined empirically. At higher values (e.g. ≥ 2.0×) genes with known expression in these cells were excluded (e.g. *acr-2, unc-5*) [14,15] (Additional file 14) whereas, a lower threshold of 1.5× included significantly more false positives (e.g. muscle genes, *pat-3, sup-10*) [16,17].

### Confirmation of UNC-4 motor neuron genes

Information gleaned from published literature  and from wormbase , identified 27 genes with known expression in embryonic motor neurons that also express *unc-4*::GFP (I5, SAB, DA) (Additional file 11). We detect 21 (78%) of these genes as EGs of which 10 (37%) are enriched. In addition, a significant number of transcripts encoding core neuronal functions (e.g. axon guidance, neurotransmitter signaling, etc.) are detected in the *unc-4*::GFP data set (Table 2, Additional file 14). For example, in addition to UNC-64 (syntaxin or t-SNARE,) other components of the SNARE complex, SNB-1 (synaptobrevin or v-SNARE) and SNAP-25 (Y22F5A.3) are detected [18,19]. We also examined the data set for potential false positives by considering transcripts that are known to be highly expressed in other tissues but not in UNC-4 motor neurons. For example, in the embryo, the homeodomain protein UNC-30 is exclusively detected in DD motor neurons. Expression of the GABA vesicular transporter, UNC-47, in DD motor neurons depends on *unc-30 *function [20]. UNC-4 motor neurons are cholinergic and as expected neither of these GABA specific transcripts are present in the *unc-4*::GFP motor neuron data set (Additional file 7).

The strong representation of ~80% of genes known to be expressed in I5, SAB, and DA motor neurons in the *unc-4*::GFP motor neuron dataset indicates that other previously uncharacterized transcripts in this list are also likely to be expressed in these cells *in vivo*. To test this idea, we evaluated GFP reporter lines for representative genes detected as enriched in the *unc-4*::GFP motor neuron data set (Fig. 5). As shown in Table 1, 82% (15/18) of these promoter-GFP fusions show expression in UNC-4 motor neurons *in vivo*. Of the GFP reporters not detected in these neurons, one of them, T19C4.5, fails to express GFP in any cell. This finding could mean that the upstream sequence selected for this construct does not overlap the endogenous T19C4.5 gene regulatory region. In some cases, cell-specific expression of *C. elegans *genes depends on distal upstream regions, intronic sequences, or 3' domains that would not be included in these 5' promoter GFP fusions [21]. This explanation could also account for the apparent absence of GFP expression in the *unc-4*::GFP motor neurons of the *nlp-9 *and *nlp-15 *GFP reporters. The validity of this data set is further substantiated by the observation that GFP expression in DA motor neurons is detected even for lower ranking genes (e.g. *syg-1*::GFP, statistical rank = 877). Thus, we believe that the transcripts listed in the *unc-4*::GFP motor neuron data set are likely to constitute an accurate representation of genes normally expressed in these cells.

We note that the positive GFP reporters shown in Table 1 are not uniformly detected in UNC-4 neurons: all but one (*flp-13*) are expressed in the DAs, one in I5 and none in the SAB motor neurons. This bias reflects the relative abundance of DA motor neurons (~70% or 9/13 of *unc-4*::GFP neurons *in vivo*) in the cells used to generate this data set and thus could indicate that most of the enriched transcripts are also expressed in the DAs. Therefore, results presented below are largely focused on potential gene functions in DA motor neurons.

### Families of neuronal genes expressed in UNC-4 motor neurons

Here we describe transcripts detected in the *unc-4*::GFP dataset with an emphasis on genes that are enriched in these cells and therefore likely to encode proteins with important roles in the differentiation or function of UNC-4 motor neurons (Table 2). A comprehensive discussion of gene families from this list can be found in Additional file 16. Selected examples are presented here. Gene names for enriched trancripts discussed in this section are shown in bold and are listed in Table 2. All EGs are listed in Additional file 7.

### Axon guidance and outgrowth

**Figure 6:**
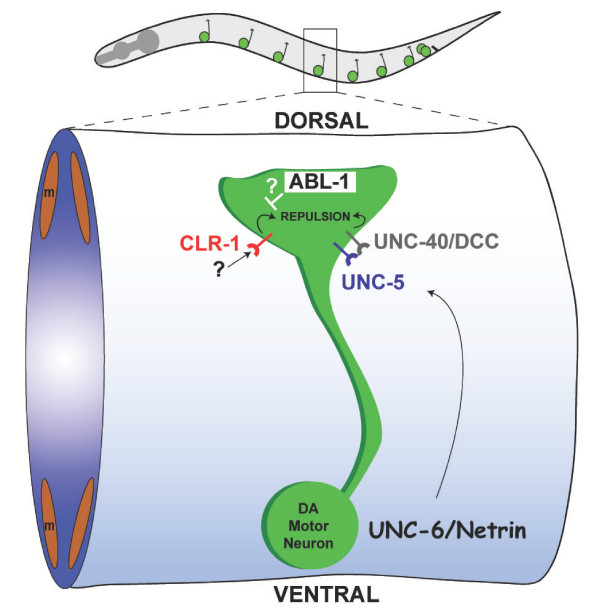
**Model of DA motor neuron axon guidance**. Ventrally derived UNC-6/Netrin guidance cues binds to the UNC-40/DCC and UNC-5 receptor to steer the DA motor axon toward the dorsal nerve cord. The receptor tyrosine phosphatase, CLR-1, promotes dorsal motor axon outgrowth via an UNC-40/DCC independent pathway [24]. The transcript encoding the *C. elegans *ortholog of Abelson tyrosine kinase (ABL-1) is enriched in the *unc-4*::GFP motor neuron data set and is proposed to antagonize CLR-1 activity.

Growth cone steering and cell migration along the dorsal-ventral body axis in *C. elegans *depend on the **UNC-6/netrin **guidance cue. The **UNC-40/DCC **receptor mediates an attractive response to **UNC-6/netrin **whereas co-expression of **UNC-40/DCC **with a second **UNC-6 **receptor, **UNC-5**, results in repulsion [15,22]. The **UNC-6/netrin **signal is released from ventral ectoderm [23] to repel growth cones expressing both **UNC-40 **and **UNC-5**; this interaction is required for normal outgrowth of DA motor neuron commissures to the dorsal nerve cord [15]. As expected, ***unc-5 ***and ***unc-40 ***transcripts are enriched in UNC-4 motor neurons. ***unc-6***, which is known to be expressed in the I5 pharyngeal neuron, is also elevated [23]. The **CLR-1 **receptor protein tyrosine phosphatase (RPTP) is proposed to inhibit attractive **UNC-6/netrin **responses via interactions with **UNC-40**. In the DA motor neurons, **CLR-1 **also promotes **UNC-6/netrin **repulsion by an **UNC-40**-independent mechanism [24]. As predicted by these models, the ***clr-1 ***transcript is elevated in UNC-4 motor neurons. Relevant to this point, we note that the *C. elegans *Abelson tyrosine kinase ortholog, ***abl-1***, is also enriched. In *Drosophila*, Abl tyrosine kinase antagonizes the axon guidance role of RPTPs in motor neurons [25]. It will be interesting to determine if **ABL-1 **functions similarly in *C. elegans *and, in this case, if **ABL-1 **works in opposition to **CLR-1 **during DA motor axon outgrowth (Fig. 6).

We also detected axon guidance effectors ***unc-115 ***and *ced-10 *in our microarray array dataset. Genetic approaches have shown that ***unc-115 ***(AbLIM, actin binding protein) and *ced-10 *(Rac GTPase) are downstream effectors of **UNC-40 **signaling and presumptive links to the cytoskeleton [26,27].

Transcripts for genes with general roles in axon outgrowth are enriched in the *unc-4*::GFP motor neuron data set. These include ***unc-44 ***(ankyrin-like), ***unc-76 ***(novel) and ***unc-14 ***(RUN domain). All three of these genes are highly expressed in the *C. elegans *nervous system. ***unc-44 ***encodes multiple alternatively spliced transcripts with broad roles in axonal morphogenesis [28]. **UNC-76 **and its vertebrate homologs define a new protein class of unknown biochemical function. In *C. elegans*, ***unc-76 ***mutants show axon outgrowth and fasciculation defects [29]. ***unc-14 ***and *unc-51 *(serine/threonine kinase) mutants display similar neuronal defects with misplaced processes and enlarged abnormal varicosities [30]. UNC-51 (EG) has been proposed to phosphorylate **UNC-14 **to regulate vesicular trafficking during axonal process outgrowth [31,32].

### Wingless signaling

Wingless (Wnt) signaling controls multiple developmental processes in the nervous system ranging from cell determination to axon guidance and synaptogenesis [33,34]. The *C. elegans *genome contains 5 Wnt genes and 4 Wnt receptors or Frizzled homologs [35]. One of each, ***cwn-1 ***(Wnt) and ***lin-17 ***(Frizzled), are enriched. Transcripts for other components of the canonical (*mig-5*, *mom-5*, *cwn-2*, *dsh-1*, *dsh-2*, *Y73B6BL.21*) and noncanonical (*lit-1*, *mom-4*, *par-1*, *tap-1*) Wnt signaling pathways are detected as EGs. Thus, UNC-4 motor neurons are presumptively competent to send as well as respond to Wnt signals. Functions for Wnt signaling in the *C. elegans *motor neuron circuit have not been defined. Possibilities include the regulation of synaptogenesis as suggested by studies of *Drosophila *motor neurons which secrete Wnt to control both presynaptic and postsynaptic differentiation at the neuromuscular synapse [36]. A gradient of Wnt signaling controls cell migration along the AP axis in *C. elegans *[37]. Responsiveness to this graded Wnt signal could account for the anterior polarity of DA motor neurons in the dorsal nerve cord as suggested by the recent finding that commissural axonal polarity along the AP axis in the vertebrate spinal cord is dependent on Wnt signaling [38].

### Nicotinic Acetylcholine Receptors (nAChRs)

The *C. elegans *genome encodes at least 27 distinct nAChR subunits [39]. Two of these, **ACR-2 **and **UNC-63 **are expressed in DA class motor neurons [14,40] and are enriched in the *unc-4*::GFP motor neuron data set. Expression of *unc-29 *[41] and *unc-38 *(J.L. Bessereau, personal communication) in ventral cord motor neurons has been previously reported and these are also detected as EGs (Additional file 7). *acr-12*::GFP is expressed in neurons (A. Gottschalk and W. Schafer, personal communication), and we have validated the enrichment of ***acr-14 ***with GFP reporters that confirm expression in DA motor neurons (Fig 5). In body muscle, **UNC-63 **is an essential component of a levamisole-sensitive nACh receptor that also includes UNC-29, UNC-38, LEV-1 and LEV-8 [40,42]. **ACR-12 **may coassemble with **UNC-63**, UNC-29, and UNC-38 to generate a related nACh receptor in UNC-4 motor neurons (A. Gottschalk and W. Schafer, personal communication). Five additional sets of nAChR subunits are detected as EGs and a so-called "orphan" ligand gated ion channel (LGIC) subunit, **F21A3.7**, with significant similarity to the nAChR gene family, is enriched. Despite the diversity of nAChR subunits expressed in UNC-4 motor neurons and the potentially complex array of resultant receptors, no functions have been directly assigned to nAChRs in these cells [43]. Although loss-of-function mutations in nAChR subunits that are also expressed in muscle (i.e. *unc-29*, *unc-3*8, ***unc-63***) result in locomotory defects, gene knockouts of ***acr-2 ***(Y. Jin, personal communication) and ***acr-12 ***(data not shown), which are exclusively expressed in neurons, do not produce obvious effects on motility or behavior. Perhaps the surprisingly large number (12) of nAChR subunit genes detected in these cells results in overlapping functions that mask defects in single gene knockout mutants. Alternatively, these nAChRs may mediate subtle aspects of motor neuron activity. This idea is consistent with models in which nAChRs act presynaptically to modulate neurotransmitter release [44,45]. Finally, we detect enrichment of transcripts for proteins **RIC-3 **(novel) and **LEV-10 **(CUB domain) that mediate nAChR localization [46,47].

### Ligand-Gated Ion Channels

UNC-4 motor neurons are potentially responsive to additional classes of neurotransmitters. Enrichment of ***glr-5 ***(kainate type ionotropic glutamate receptor subunit) is correlated with its known expression in the SAB motor neurons [48]. As members of the GABA/Glycine family of ligand-gated receptors, the presumptive anion channels encoded by **T27E9.9 **and **Y71D11A.5 **are predicted to hyperpolarize UNC-4 motor neurons and thus inhibit cholinergic activity [49]. It may be significant that a candidate sodium/chloride-dependent glycine transporter, ***snf-5***, is enriched. (**C09E8.1**, an outlier in the sodium/chloride-dependent transporter family is also enriched.) In mammalian cells, plasma membrane transporters GLYT1/GLYT2 remove glycine from the synaptic cleft, and in the case of GLYT2, thereby recycle glycine for reuptake into synaptic vesicles [50]. UNC-4 motor neurons do not express the GABA/Glycine vesicular transporter, UNC-47, however, and are therefore unlikely to release glycine presynaptically [51]. In this case, the physiological function of the **SNF-5 **transporter could mirror that of GLYT1, which is believed to attenuate glycinergic signaling by pumping glycine into a non-glycinergic glial cell [52]. To date, the potential function of glycinergic signaling in *C. elegans *has not been explored.

### G-protein signaling

**Figure 7:**
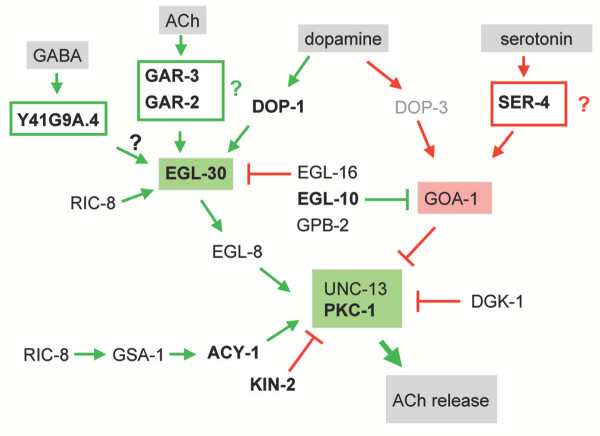
**G-protein signaling pathways regulating neurotransmitter release in cholinergic motor neurons**. Components shown in bold are enriched in the *unc-4*::GFP motor neuron data set. All others are EGs with the exception of the *dop-3 *transcript which is not detected (light gray text). See Table 3 for protein descriptions. Green denotes interactions that promote acetylcholine (ACh) release and red marks steps that inhibit synaptic vesicle fusion. Neurotransmitters are highlighted in gray boxes. This figure adapted from Reynolds et al. (2004) [60].

Cholinergic motor neuron activity in *C. elegans *is modulated by G-protein signaling pathways that respond to the neurotransmitters acetylcholine, serotonin (5-HT), and dopamine (Fig. 7) [53-55]. In each case, acetylcholine release is either promoted by **EGL-30 **(Gα_q_) or inhibited by GOA-1 (Gα_o_). Input to these antagonistic pathways is provided by G-protein coupled receptors (GPCRs). Pharmacological evidence suggests that a muscarinic acetylcholine receptor activates **EGL-30 **to enhance ACh release at the neuromuscular synapse [53,56]. The enriched muscarinic AChRs, **GAR-2 **and **GAR-3 **could account for this effect [57,58]. Similarly the enriched 5-HT receptor, **SER-4**, is a strong candidate for the GPCR mediating the inhibitory effect of serotonin on ACh release from ventral cord motor neurons [54]. Dopamine may either activate or inhibit ACh release within the same cholinergic motor neuron. Activation depends on **DOP-1 **which is enriched in UNC-4 motor neurons. Inhibition is attributed to DOP-3. Expression of DOP-3 in cholinergic ventral cord motor neurons is reportedly weak and we do not detect the *dop-3 *transcript in our data set [55]. UNC-4 motor neurons are also potentially responsive to GABA as a transcript (**Y41G9A.4**) encoding a metabotropic GABA type B1 receptor is enriched. GABA dependent effects on cholinergic motor neuron activity have not been previously reported in *C. elegans*.

Genetic screens for mutations affecting neurotransmitter release have revealed a complex web of interacting components that couple G-protein signaling to synaptic vesicle fusion (Fig 7) [53,56,59,60] (D. Sieburth and J. Kaplan, personal communication). With one exception (*dop-3*), transcripts for all of the known components of these pathways are either enriched (***acy-1***, ***egl-10***, ***gpc-2***, ***kin-2***, ***pkc-1***) or detected as EGs (*dgk-1*, *egl-8*, *egl-16*, *gpb-2*, *gsa-1*, *kin-2*, *ric-8*, *unc-13*). Lack of enrichment of some of these components is consistent with the widespread utilization of G-protein signaling pathways in *C. elegans *neurons and muscle cells [61,62]. As noted above, these data have also revealed several additional enriched transcripts with potential roles in G-protein dependent locomotory behavior. ***egl-47 ***encodes an orphan GPCR and ***rgs-1 ***an RGS protein, both of which can regulate *goa-1 *signaling in the egg laying circuit [63,64]. RNAi of **F39B2.8**, which encodes a highly conserved but unusual protein with both serine/threonine kinase and 7-transmembrane domains, results in a locomotory defect [65] that could be indicative of a neuromodulatory function in DA motor neurons. A complete list of G-protein signaling components detected in this dataset can be found in Table 3.

**Table 3:** Summary of G-protein signaling genes in *unc-4::*GFP neurons. Genes either known to function in *C. elegans *motor neuron G-protein signaling pathways or likely to have a role therein (e.g. SER-4) are listed here with KOG descriptions. Transcripts are listed as either enriched with statistical rank (top) or as EGs (bottom).

**Cosmid Name**	**Common Name**	**Statistical Rank**	**KOG Description**
			
***Enriched***			
F47D12.1	*gar-2*	245	7 transmembrane receptor
Y40H4A.1	*gar-3*	421	Muscarinic acetylcholine receptor
F15A8.5d	*dop-1*	683	7 transmembrane receptor
Y22D7AR.13	*ser-4*	680	7 transmembrane receptor
Y41G9A.4		769	GABA-B ion channel receptor subunit GABABR1 and related subunits, G-protein coupled receptor superfamily
M01D7.7a	*egl-30*	124	G protein subunit Galphaq/Galphay, small G protein superfamily
F28C1.2	*egl-10*	872	G protein signaling regulators
F57F5.5	*pkc-1*	743	Serine/threonine protein kinase
F17C8.1	*acy-1*	884	Adenylyl cyclase
R07E4.6	*kin-2*	467	cAMP-dependent protein kinase types I and II, regulatory subunit

***Present (EGs)***			
			
Y69A2AR.1	*ric-8*		Signaling protein RIC-8/synembryn (regulates neurotransmitter secretion)
C16C2.2a	*eat-16*		G protein signaling regulators
B0348.4a	*egl-8*		Phospholipase C
F52A8.2	*gpb-2*		G-protein beta subunit
C26C6.2	*goa-1*		G-protein alpha subunit (small G protein superfamily)
ZK542.2a	*unc-13*		Neurotransmitter release regulator, UNC-13
C09E10.2a	*dgk-1*		Diacylglycerol kinase
R06A10.2	*gsa-1*		G protein subunit Galphas, small G protein superfamily

### Neuropeptide signaling

The *C. elegans *genome includes a large and diverse array of genes encoding potential neuroactive peptides. GFP reporter studies indicate that these genes are predominantly expressed in neurons. 23 "flp" genes encoding FMRFamide and related peptides (FaRPs) have been described. FaRPs have been shown to modulate a wide array of invertebrate neural functions [66]. Previously reported expression of ***flp-2, flp-4, flp-13 ***in the pharyngeal I5 neuron [67] (Fig 5) is confirmed by their enrichment in the *unc-4*::GFP motor neuron data set (Table I). ***flp-5 ***is also elevated in these cells and 8 additional flps are detected as EGs (Additional Files 7, 14). Specific FaRPs modulate cell excitability (***flp-13***), locomotion (*flp-1*) and feeding behavior (*flp-21*) in *C. elegans *[68,69]. The inhibitory action of the **FLP-13 **peptide on pharyngeal muscle activity is consistent with its expression in I5 [70].

The *C. elegans *genome contains 37 genes encoding predicted insulin-like peptides [71]. Transcripts for two of these, ***ins-1 ***and ***ins-18***, are enriched; *ins-17*, *ins-24 *and *ins-30 *are present but not significantly elevated relative to other cells. ***ins-1 ***and ***ins-18 ***have been implicated in the DAF-2 insulin receptor dependent pathways regulating growth, metabolism and lifespan [71].

A total of 32 genes encoding other potential classes of neuropeptides have also been identified in the *C. elegans *genome. Three of these neuropeptide-like protein (nlp) genes, ***nlp-9***, ***nlp-15***, and ***nlp-21***, are enriched in UNC-4 motor neurons (Table 2). An additional group of 11 nlp transcripts are detected as EGs (Additional File 7, 14). To date, no functions have been directly assigned to nlp genes in *C. elegans *[72].

Our studies have revealed that a surprisingly large number of neuropeptide genes are transcribed in UNC-4 motor neurons. These results indicate that UNC-4 motor neurons are likely to exhibit significant neurosecretory activity. This conclusion is consistent with our finding that proteases required for neuropeptide processing and activation [**T03D8.3 (**Proprotein convertase (PC) 2 chaperone), *egl-3 *(zinc carboxypeptidase) and *egl-21 *(subtilisin-like proprotein convertase)] are also expressed in these cells [73-75]. Other genes with important roles in neurosecretion are also detected. ***ric-19 ***encodes a novel arfaptin-related protein that is believed to function in the Golgi in the generation of neurosecretory granules and may through this activity and subsequent neuropeptide signaling exert an indirect effect on ACh release from conventional synaptic vesicles [76,77]. Our finding that ***ric-19 ***is highly enriched in cholinergic motor neurons could be indicative of autocrine neuropeptide modulation of ACh secretion at the neuromuscular synapse. Consistent with this idea is our finding that *ida-1*, a conserved membrane component of the dense core vesicles in which neuropeptides are typically packaged, is an EG [78]. Finally, **UNC-31 **(CAPS), a known facilitator of dense core vesicular release, is enriched [78]. Plasma membrane fusion of both dense core vesicles and the small, clear vesicles in which classical neurotransmitters are packaged, depend on a common set of calcium-activated components [79] most of which are either enriched or present in these cells (see Table 2 and Additional Files 14, 16).

In addition to secreting neuropeptides, UNC-4 motor neurons are also likely to respond to neuropeptide signaling. Transcripts for nine putative neuropeptide receptors are enriched. (Table 2). RNAi of two of these, ***npr-2 ***and **F59D12.1**, results in locomotory defects that could be indicative of specific functions in DA motor neurons [65]. ***npr-2 ***is a close relative of *npr-1 *(not detected) which has been shown to control social feeding behavior in response to the FLP-21 (not detected) peptide [69]. **F59D12.1 **is most closely related to melatonin receptors but its *in vivo *ligand is unknown. Neuropeptides are believed to modulate secretion of classical neurotransmitters [79]. Neuropeptide specific effects on excitatory motor neuron activity in the *Ascaris *ventral nerve cord have been reported [80]. Genetic evidence in *C. elegans *indicates that acetylcholine release at the neuromuscular junction is enhanced by neuropeptide activity [73] and that this pathway depends on the EGL-30 G_q_α protein [68] (Table 2). These neuropeptides may be released from neurons and also as a retrograde signal from muscle cells [73,81].

Other classes of enriched transcripts are discussed in Additional file 16 (Transcription factors, Cell Adhesion Molecules, Synapse-Associated Proteins, Neurotransmitter Vesicular Release Components, TGF-β Signaling Proteins, Serpentine Receptors, Calcium Channels, Calcium Ion Binding Proteins, Potassium Channels, Innexins, DEG/ENaC Channels, and Stomatins).

## Discussion

We have described MAPCeL, a microarray-based strategy for fingerprinting specific *C. elegans *neurons, and provide evidence that this approach can reveal a comprehensive picture of gene expression in these cells *in vivo*. *unc-4*::GFP-marked neurons were isolated by FACS from primary cultures of embryonic cells and profiled on the *C. elegans *Affymetrix gene chip. Because these *unc-4*::GFP neurons differentiate *in vitro*, it was important to establish that our microarray data provide an accurate representation of gene expression in the intact animal. This conclusion is supported by three observations: (1) A majority (21/27) of genes with known expression in *unc-4*::GFP neurons are detected in our microarray data set; (2) ~80% (15/18) of GFP reporters constructed for transcripts enriched in UNC-4 motor neurons are expressed in these cells *in vivo *(Table 1, Fig. 5); (3) Transcripts known to encode proteins with key roles in *unc-4*::GFP motor neuron differentiation (e.g. axon guidance and outgrowth, synaptogenesis) and function (e.g., neurotransmitter vesicle release, G-protein signaling pathways) are highly represented in our data sets. These findings parallel earlier studies showing that cultured *C. elegans *neurons and muscle cells adopt apparently normal morphological and physiological characteristics [6] and are consistent with evidence favoring a cell autonomous mode of differentiation for *C. elegans *embryonic cells after an initial phase of inductive signaling events [4,82]. We have now generated comparable microarray profiles of other motor neuron classes and muscle cells that also show strong congruence with known patterns of gene expression (RMF, SEV, SJB, DMM, unpublished data). We therefore conclude that our approach of profiling GFP marked neurons isolated from primary culture can now be widely applied to fingerprint specific *C. elegans *embryonic cells. In some cases, however, differentiation of a given neuron is likely to depend on specific intercellular signals that primary cultures will not provide. Thus, in every instance, it will be necessary to confirm microarray data by independent methods as described here.

### Methods for profiling specific *C. elegans *cells

Previous studies have described other methods for cataloging transcripts from specific *C. elegans *cells. Comparisons of microarray data from mutant animals with either supernumerary or absent sensory neurons in the male tail, have revealed genes that are preferentially expressed in these cells [83]. However this approach is limited to cell types that can be manipulated by specific mutants. In addition, this method may be insufficiently sensitive to detect changes in smaller subsets of cells due to high background mRNA from cells that are not affected by the mutation (SEV, DMM, unpublished data). This limitation can be overcome by enriching for mRNA from target cells. To this end, Zhang et al. (2002) used an approach similar to the strategy outlined in this paper to identify downstream genes of the MEC-3 transcription factor in *C. elegans *touch neurons [84]. However, this work did not provide a comprehensive cell-specific gene expression profile as we have here perhaps due to the limited enrichment (~50%) of GFP-labeled touch neurons. We have now optimized the application of nematode embryonic cell culture and FACS technology to obtain ~90% enrichment of GFP-marked neurons and muscle cells (Fig. 2) (RMF, SEV, SJB, DMM, unpublished data). These methods have now been successfully applied to profile other classes of *C. elegans *embryonic cells [85,86].

MAPCeL cannot be used for postembryonic cells because these apparently do not arise in culture [6]. Microarray profiles of specific larval cells have been obtained, however, by mRNA tagging. In this approach, an epitope-labeled polyA binding protein (FLAG-PAB-1) is expressed transgenically under the control of a cell-specific promoter and mRNAs isolated by co-immunoprecipitation with anti-FLAG. This method has been used for microarray analysis of *C. elegans *body muscle cells and ciliated sensory neurons [87,88]. We have now successfully used the mRNA tagging strategy to profile specific subsets of motor neurons from *C. elegans *larvae (SEV, RMF, J. Watson, S. Kim, P. Roy, DMM, unpublished data). Thus, in principle, it should now be possible to obtain an accurate gene expression profile for virtually any *C. elegans *cell throughout development.

### UNC-4 motor neurons are sensitive to a wide range of neurotransmitters and peptidergic signals

**Figure 8:**
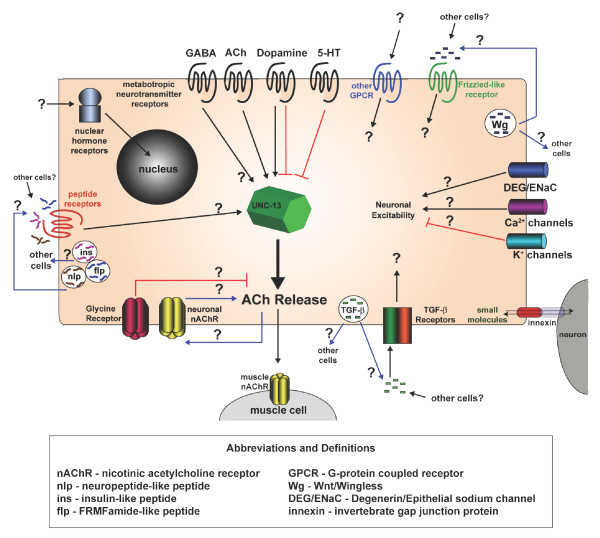
Signaling components detected in *unc-4*::GFP motor neurons.

Acetylcholine (ACh) release at the DA neuromuscular junction is presumptively triggered by excitatory input from command interneurons. The strength of the DA cholinergic signal, however, may be strongly modulated by other cells that release neurotransmitters from distal locations. For example, dopamine is produced by 8 neurons, none of which are presynaptic to DA motor neurons [89]. Dopamine, however, is a potent regulator of cholinergic secretory activity in the ventral motor circuit. The dopamine effect is mediated in part by DOP-1, a G-protein coupled receptor (GPCR) [55]. We have confirmed enrichment of the *dop-1 *transcript and also detected elevated levels of transcripts encoding GPCRs for acetylcholine and serotonin, additional neurotransmitters known to modulate cholinergic motor neuron activity via G-protein signaling pathways [18,55]. Enrichment of a GABA metabotropic receptor transcript offers yet another mechanism for exogenous adjustment of neurotransmitter vesicular fusion in DA motor neurons. Indirect evidence indicates that acetylcholine release from ventral cord motor neurons may also be sensitive to neuropeptide signals from other neurons or muscle cells [73,81]. We have established that *unc-4*::GFP motor neurons express elevated transcript levels for nine different GPCRs with significant homology to insect or mammalian neuropeptide receptors. This signaling complexity is further compounded by the enrichment of transcripts for 18 members of the serpentine GPCR-like family in *unc-4*::GFP neurons (Additional Files 9, 16). Ligands for this outlier group of GPCRs are unknown [90]. The picture emerging from these data is of a motor neuron festooned with multiple G-protein linked receptors each responding to a different class of neurotransmitter or peptidergic signal (Fig 8). In effect, these motor neurons are also functioning as a kind of sensory neuron in which disparate inputs are internally assessed to fine-tune output in concert with temporal requirements for locomotory activity.

The microarray data also reveal multiple additional classes of receptors and ion channels through which the differentiation and function of *unc-4*::GFP motor neurons could be modulated by extracellular signals (Fig 8). Finally, we have detected enrichment of transcripts encoding TGF-β, wingless, and several classes of neuropeptides (Table 2, Additional Files 9, 16). Thus, in addition to responding to a wide range of stimuli, *unc-4*::GFP motor neurons are also potentially capable of regulating the activities of other cells with a variety of different signals. If an organism as simple as *C. elegans *builds motor neurons with such sophisticated signaling and response mechanisms, it is tempting to speculate that neurons in other, more advanced species may have evolved even more complex pathways. It is likely, however, that the core signaling systems described here are also conserved. This prediction is underscored by our finding that approximately half of the enriched transcripts (537/1012) and 2/3 of EGs (4050/6217) detected in *unc-4*::GFP neurons have human homologs (BLAST ≤ e^-10^) (Additional Files 12, 13).

### Applications of MAPCeL

In addition to confirming expression of genes with known roles in *unc-4*::GFP motor neuron differentiation and function, the microarray data also uncovered strong candidates for new genes governing these events. For example, DA motor axons grow dorsally in response to a ventrally provided repulsive UNC-6/netrin guidance cue [15]. Recent work has shown that the receptor protein tyrosine phosphatase (RPTP), CLR-1, positively enhances this response [24]. As expected, we found that the *clr-1 *transcript is enriched in the *unc-4*::GFP motor neuron data set. We also noted enrichment of *abl-1*, the *C. elegans *homolog of Abelson tyrosine kinase. By analogy to findings in *Drosophila *in which Abelson tyrosine kinase functions in opposition to RPTP-dependent axon guidance [91], we propose that ABL-1 antagonizes CLR-1 activity (Fig 6). This model predicts that either genetic ablation or RNAi of *abl-1 *will suppress the DA motor axon guidance defects of *clr-1 *mutants.

Another application of this strategy includes the identification of transcription factor target genes. A comparison of expression fingerprints of wildtype cells vs cells that are mutant for a specific transcription factor could reveal downstream genes [84]. For example, the UNC-4 transcription factor regulates axon morphology and synaptic strength in embryonically derived *unc-4*::GFP neurons [8]. UNC-4 also defines the specificity of synaptic inputs to postembryonically-derived VA motor neurons [7,92,93]. We have now used a combination of MAPCeL and mRNA tagging strategies to identify candidate genes regulated by UNC-4 in these cells (RMF, SEV, DMM, unpublished data). Gene regulatory motifs to which transcription factors bind may also be revealed as common cis-acting sequences in cohorts of co-regulated genes [94].

The *C. elegans *nervous system is composed of exactly 302 neurons. The morphology and connectivity for every neuron has been defined by serial section electron microscopy to generate a detailed wiring diagram for the entire network [2]. The *C. elegans *genome is similarly well defined. All 6 chromosomes are completely sequenced and the structure of over 20,000 genes described [5]. Unique combinations of these genes are likely to specify different classes of neurons and their differentiated traits. The problem now is to link the gene map with the wiring diagram. We believe that MAPCeL offers a powerful approach toward achieving this goal.

## Conclusion

We have described a new method, Micro-Array Profiling of *C. elegans *cells, or MAPCeL, for generating gene expression fingerprints of subsets of *C. elegans *neurons. Embryonic motor neurons marked with a reporter gene, *unc-4*::GFP, were isolated by FACS from primary cultures and profiled on the *C. elegans *Affymetrix array. We confirmed that microarray data generated by this approach reliably identify genes expressed by these motor neurons *in vivo*. We propose that MAPCeL can now be used to generate a gene expression map for the *C. elegans *nervous system.

## Methods

### Cell Culture

Embryonic cells were obtained using methods previously described [6]. Briefly, embryos were isolated from gravid adults following lysis in a hypochlorite solution. Intact embryos were separated from debris by flotation on 30% sucrose. Eggshells were removed by incubation in 0.5 ml chitinase (0.5 U/ml in egg buffer) for 45 minutes. Following resuspension in L-15 medium supplemented with 10% FBS (L15-10) and antibiotics, the embryos were dissociated by passage through a 5μm syringe filter (Durapore). Cells were plated on poly-L-lysine (0.01%, Sigma) coated single-well chambered coverglasses (Nalge Nunc International) at a density of ~10 million cells/ml and maintained in L15-10 media. Cells were incubated at 25°C in a humidified chamber. Wildtype (N2) cells were isolated and treated similarly.

### FACS analysis

Sorting experiments were performed on a FACStar Plus flow cytometer (Becton Dickinson, San Jose, CA) equipped with a 488 nm argon laser. Emission filters were 530 ± 30 nm for GFP fluorescence and 585 ± 22 nm for PI fluorescence. The machine was flushed with egg buffer [6] prior to sorting to enhance viability. 2 μm fluorescent beads were used to calibrate light scattering parameters for the relatively small size of *C. elegans *embryonic cells. Cells were sorted at a rate of 4000–5000 cells per second through a 70 μm nozzle.

Immediately prior to sorting, supernatant from the 24 hour cultures was removed and discarded. 1 ml of egg buffer was added to the chamber coverglass. Cells are loosely adherent to poly-L-lysine and can be easily dislodged with gentle pipetting. 3 ml of egg buffer + cells were drawn into a 3 cc syringe and the suspension filtered with a 5 μm Durapore syringe filter. Propidium Iodide (PI) was added to the cell suspension at a final concentration of 5 μg/ml prior to sorting. Autofluorescence levels were established by flow cytometry of cells isolated from wildtype (i.e. non-GFP) embryos (See Fig 2A). Next, wildtype cells stained with PI were used to define the sorting gate for damaged cells. GFP+ cells containing no PI were sorted to establish the intensity range of GFP fluorescence. Finally, *unc-4*::GFP cells stained with PI were gated (Fig 2B) using the parameters established above. The sorting gate for size and granularity (Fig 2C) was empirically adjusted to exclude cell clumps and debris and to achieve ~90% enrichment for GFP-labeled cells. *unc-4*::GFP cells were collected in a 15 ml conical tube containing 1 ml of L15-10 media. Cells were pelleted using low-speed cenrifugation (300 × g) and either plated on peanut lectin-coated slides for visualization [6] or used for RNA isolation (see below). Reference cells were obtained from 1 day old cultures of embryonic blastomeres isolated from the non-GFP wildtype strain (N2). In this case, all viable cells (i.e. non-PI stained) were collected by FACS for RNA isolation.

### RNA isolation, amplification, and hybridization

RNA was prepared from FACS-isolated *unc-4*::GFP cells for comparison to RNA from the wildtype reference strain (N2). Cells were pelleted using low-speed centrifugation (300 × g). The supernatant was removed and RNA was extracted with a micro-RNA isolation kit (Stratagene) using the recommended volumes for 1 million cells. Typical yields were 1 pg total RNA/cell. 100 ng of total RNA was subjected to 2 rounds of amplification, as described in the Affymetrix GeneChip Eukaryotic Small Sample Target Labelling Protocol, with the following modifications. 100 ng (5 pmol) of T7-dT primer (5'-GGCCAGTGAATTGTAATAC GACTCACTATAGGGAGGCGG-(dT)_24_-3') was used as opposed to the recommended 100 pmol. RNA cleanup was achieved using the RNeasy mini kit (Qiagen); 300 μl of 100% ethanol (final concentration = 40% ethanol) was added to the sample prior to absorption to the column matrix. Eluate was passed through the column 2× prior to washing to improve yields. The BioArray High Yield RNA Transcript Labeling Kit (Enzo) was used to biotinylate the sample in the second round of amplification. 10–15 μg of labeled aRNA (amplified RNA) was fragmented and hybridized to the Affymetrix *C. elegans *chip according to the Affymetrix Expression Analysis Technical Manual. The Agilent Bioanalyzer was used to assess RNA quality prior to labeling and to confirm fragmentation (<200 bp) before hybridization.

### Data analysis

The commercially available *C. elegans *Affymetrix array was used for all experiments. This chip was designed using the December 2000 genome sequence. All probe set information is available at  as well as . *unc-4*::GFP neurons were profiled in triplicate; baseline data (all cells) were obtained from four independent experiments with wildtype embryonic cells. Hybridization intensities for each experiment were scaled in comparison to a global average signal from the same array (A complete list of Affy normalized values can be found in Additional file 2) [95]. Expressed transcripts were initially identified on the basis of a "Present" call in a majority of experiments (2/3 for *unc-4*::GFP and 3/4 for wildtype cells) as determined by Affymetrix MAS 5.0 (see below) (Additional Files 4, 5). In this approach, a Mismatch (MM) value for each feature is compared to a Perfect Match (PM) value to estimate non-specific binding. This strategy, however, tends to arbitrarily exclude low intensity signals in which PM and MM values may be comparable [96,97]. To avoid this bias in the detection of transcripts that might be differentially elevated in the *unc-4*::GFP data set, intensity values were normalized using RMA (Robust Multi-Array Analysis) available through GeneTraffic (Iobion) in which the MM values are not considered (Additional file 3) [96,97]. Comparisons of RMA normalized intensities for *unc-4*::GFP vs reference cells were statistically analyzed using Significance Analysis of Microarrays software (SAM, Stanford) [98,99]. A two-class unpaired analysis of the data was performed to identify genes that differ by ≥ 1.7-fold from the wildtype reference at a False Discovery Rate (FDR) of ≤ 1%. These genes were considered significantly enriched (Additional file 9). This analysis also identified ~1600 transcripts that are depleted (1.7×, ≤ 1% FDR) in *unc-4*::GFP cells vs the wildtype reference (Additional file 10). Although 729 of these transcripts are also scored as "present" in the *unc-4*::GFP motor neuron dataset, we attribute their detection to high expression in the small fraction (~10%) of non-GFP cells contaminating this preparation (see above). Therefore, we excluded all 729 of these wildtype-enriched transcripts from the list of present calls in the *unc-4*::GFP motor neuron data set (Additional file 6). Finally, to compute the overall sum of Expressed Genes (EGs) in the *unc-4*::GFP data set we restored 118 *unc-4::*GFP-enriched genes that were initially excluded from the present list due to high mismatch signals. These considerations produce a final list of 6,217 genes that are detected in *unc-4*::GFP motor neurons (Additional file 7). (see Logic Tree, Additional file 17).

### Annotation of datasets

A Wormbase mirror was established by downloading code and databases from . Using the acedb perl module, an annotation script was generated that queries the wormbase mirror. Affymetrix IDs have been mapped to specific transcripts in wormbase. Text files containing Affy IDs (one per line) and cosmid names are input into the script which then searches the wormbase mirror and matches Affy ID/cosmid name to a specific transcript. Cosmid names are used for this search when Affy IDs have not been mapped in wormbase. This information is used to acquire other linked annotations (i.e. KOG, common name, RNAi phenotype, Expression data, Kim mountain data and Gene Ontology, etc.).

### *In litero *analysis

An extensive literature search was performed using Textpresso . The keywords "DA motor neurons" generated a list of 68 citations, a similar search was conducted using the keywords "I5 pharyngeal neuron" and "SAB neurons" that detected an additional 21 citations. Expression patterns on wormbase were also searched using the "Cell identity" function to identify genes with documented expression in DA, SAB or I5. A list of 27 genes with documented expression in DA motor neurons, the I5 pharyngeal neuron and the SAB neurons was compiled from this information (Additional file 11).

### Strains

Nematode strains were maintained at 20–25°C using standard culture methods [100]. The wildtype strain was N2. Transgenic lines carrying promoter GFP fusions are listed in Additional file 1.

### Generating transgenic promoter GFP strains

*twk-30*::GFP (25 ng/ul) was microinjected with the *myo-3*::dsRed2 marker (25 ng/ul) [101]. Other transgenics were generated by biolistic transformation with promoter::GFP constructs from the Promoterome project (Additional file 1). Primer sequences for "promoterome" constructs can be found at [102]. Microparticle bombardment was conducted [103] in a BioRad Biolistic PDS-1000/He equipped with the Hepta Adapter. Gold beads (1 micron) were coated with DNA at 1 ug/ul. 100 mm NGM plates were seeded with a monolayer of ~100,000 L4/adult *unc-119 (ed3) *animals. For each construct, 1 'shot' was performed using a 1550 psi rupture disk at 28 inches of Hg vacuum. After a 1 hr recovery period, animals were washed from the plates with 7 ml M9 buffer and transferred to 7 NGM plates (1 ml/plate). Animals were grown at 20°C for 1 week. To pick transgenic animals, one-half of the plate was 'chunked' and added to a new 100 mm NGM plate; animals with wildtype movement were picked to 60 mm NGM plates and allowed to self. Worms derived from separate plates were considered independent lines; at least 2 lines were obtained for each construct.

### Microscopy

Transgenic animals and cultured cells were visualized by differential interference contrast (DIC), or epifluorescence microscopy using either a Zeiss Axioplan or Axiovert compound microscopes. Images were recorded with CCD cameras (ORCA I, ORCA ER, Hamamatsu Corporation, Bridgewater, NJ). Some images were recorded on a Zeiss 510 META confocal microscope.

## Authors' contributions

RMF developed the FACS protocol, RNA amplification methods, performed *unc-4*::GFP microarray experiments, analyzed microarray data, generated/scored GFP reporters, and helped draft the manuscript. SEV helped develop RNA amplification and bombardment protocols, generated/scored GFP reporters and helped draft the manuscript. SJB generated the reference dataset. CS and KO wrote Perl scripts used to annotate datasets, make comparisons, and identify Present genes. JM offered advice on statistical methods. DD and MV provided unpublished GFP reporter constructs as part of the Promoterome project. DMM collected images of GFP lines in the confocal microscope, oversaw all aspects of the project and helped draft the manuscript.

## Supplementary Material

Additional File 1 ***promoter*::GFP fusions **Strain names, source and references for GFP reporters used to validate microarray data. Click here for file

Additional File 2 **Intensity Values Following Global Normalization (Affymetrix MAS 5.0) **Raw intensity values were scaled against a global average intensity value calculated for each chip using Affymetrix MAS 5.0. Column A lists Affymetrix IDs for 22,548 features on the chip. These are indexed by rows with normalized intensity values for each independent hybridization and its Present/Absent (P/A) call. Columns labeled DMR 28, 30, 32, 34 include intensity values for hybridizations using RNA from all N2 (wildtype) cells. Columns labeled DM39, 51, 59 include the experimental hybridizations using RNA from FACS-isolated *unc-4*::GFP neurons. Click here for file

Additional File 3 **Intensity values after RMA normalization. **Raw data from Additional file 2 were normalized with Robust Multi-array Analysis (RMA) (GeneTraffic, version 2.8) (Iobion). Normalized values are listed for the 22,548 features on the Affymetrix *C. elegans *chip. Affy IDs are listed in column A. Columns C-I show RMA normalized values for individual replicates. DMR 28, 30, 32, 34 are the baseline hybridizations using RNA from all N2 (wildtype) cells. DM39, 51, 59 are the experimental hybridizations using RNA from FACS-isolated *unc-4*::GFP neurons. Click here for file

Additional File 4 **N2 (wildtype) Expressed Genes (EGs) **9103 genes were called "Present" by MAS 5.0 in 3 out of 4 N2 hybridizations and are listed here as EGs. Affymetrix ID, Cosmid Name and common names (Columns A-C respectively) are given for each gene. Column D contains KOG descriptions and Column E indicates the dataset in which an individual gene is present. Click here for file

Additional File 5 ***unc-4*::GFP Present Genes **6828 genes were called "Present" by MAS 5.0 in 2 out of 3 *unc-4*::GFP hybridizations and are listed here. Click here for file

Additional File 6 ***unc-4*::GFP Present Genes minus N2 Enriched Genes **The list of *unc-4*::GFP "present" calls in Additional file 5 is likely to include transcripts that are highly expressed in the small fraction of contaminating non-GFP cells (~10%). These transcripts were removed from the list of *unc-4*::GFP Present calls (Additional file 5) by subtracting genes that are highly enriched in N2 cells (Additional file 10). The balance of 6099 genes is listed here with Affy ID, Cosmid Name, Common Name and KOG description. Click here for file

Additional File 7 ***unc-4*::GFP Expressed Genes (EGs) **RMA normalization ignores the MAS 5.0 P/A calls and therefore does not discard some of the "absent" genes that are excluded from Additional file 6 (*unc-4*::GFP Present Genes). As a result, the *unc-4*::GFP enriched genes (Additional file 9) identified by SAM statistics actually includes 118 genes that are not listed in Additional Files 5 and 6. These 118 additional *unc-4*::GFP enriched genes were therefore restored to the list of *unc-4*::GFP Present Genes in Additional file 6 to provide a more accurate list of genes (6217) that are actually expressed in *unc-4*::GFP cells. These *unc-4*::GFP Expressed Genes (EGs) are listed with Affy ID, Cosmid Name, Common Name and KOG description. Click here for file

Additional File 8 **Expressed Genes (EGs) **EGs from N2 (Additional file 4) and from *unc-4*::GFP (Additional file 7) cells were combined to detect a total of 10,071 unique EGs. Click here for file

Additional File 9 ***unc-4*::GFP Enriched Genes **Transcripts elevated 1.7 fold above baseline at a False Discovery Rate (FDR) ≤1% were considered enriched. 1012 transcripts met these criteria and are listed according to statistical rank (Column A). Annotation includes Affy ID, Cosmid Name, Common Name and KOG description (Columns B-E); SAM score, Fold Change and q-value are listed (Columns F-H). We attribute the high ranking (2) of *dpy-20 *(T22B3.1) to its use as a coselectable marker in the generation of the *unc-4*::GFP transgenic line [104]. Click here for file

Additional File 10 **N2 Enriched Genes **1586 genes are enriched 1.7 fold (FDR ≤1%) in N2 cells compared to *unc-4*::GFP neurons. These are listed according to statistical rank (Column A). Annotation includes Affy ID, Cosmid Name, Common Name and KOG description (Columns B-E); SAM score, Fold Change and q-value are listed (Columns F-H). Click here for file

Additional File 11 **Genes previously known to be expressed in *unc-4*::GFP neurons. **Published literature and wormbase  were searched to identify 27 genes that are expressed in embryonic neurons that also express *unc-4*::GFP (I5, DA, SAB). Click here for file

Additional File 12 ***unc-4*::GFP motor neuron enriched genes with human homologs. **537 Enriched genes (Additional file 9) and human genes listed here have BLAST scores ≤ e^-10^. Click here for file

Additional File 13 ***unc-4*::GFP EGs with human homologs. **4050 Expressed genes (Additional file 8) and human genes listed here have BLAST scores ≤ e^-10^. Click here for file

Additional File 14 **Summary of *unc-4*::GFP enriched and expressed (EGs) transcripts in selected categories. **This file is an expanded version of Table 2 (see text) in which EGs are added to the list of enriched genes. Genes are organized as in Table 2 according to molecular function (KOG description, other description ). Statistical rank is indicated for each enriched transcript and "EG" designates transcripts that are EGs. Click here for file

Additional File 15 **Genes Expressed only in *unc-4*::GFP neurons. **Affy ID, Cosmid Name, Common Name and KOG description for 968 genes that are expressed in *unc-4*::GFP neurons. These genes are not detected in the reference (N2) dataset. Click here for file

Additional File 16 **Gene families represented in *unc-4*::GFP neurons. **A comprehensive description of neuronal transcripts organized according to gene family. Click here for file

Additional File 17 **Logic tree of data analysis methods.** Click here for file

## References

[B1] Shirasaki R, Pfaff SL (2002). Transcriptional codes and the control of neuronal identity. Annu Rev Neurosci.

[B2] White JG, Southgate E, Thomson JN, Brenner S (1986). The structure of the nervous system of the nematode Caenorhabditis elegans. Phil Trans R Soc Lond.

[B3] Sulston JE, Horvitz HR (1977). Post-embryonic cell lineages of the nematode, Caenorhabditis elegans. Dev Biol.

[B4] Sulston JE, Schierenberg E, White JG, Thomson JN (1983). The embryonic cell lineage of the nematode Caenorhabditis elegans. Developmental Biology.

[B5] Consortium TCS (1998). Genome Sequence of the nematode C. elegans: A platform for investigating biology. Science.

[B6] Christensen M, Estevez A, Yin X, Fox R, Morrison R, McDonnell M, Gleason C, Miller DM, Strange K (2002). A primary culture system for functional analysis of C. elegans neurons and muscle cells. Neuron.

[B7] Miller DMIII, Niemeyer CJ (1995). Expression of the unc-4 homeoprotein in Caenorhabditis elegans motor neurons specifies presynaptic input. Development.

[B8] Lickteig KM, Duerr JS, Frisby DL, Hall DH, Rand JB, Miller DM (2001). Regulation of neurotransmitter vesicles by the homeodomain protein UNC- 4 and its transcriptional corepressor UNC-37/groucho in Caenorhabditis elegans cholinergic motor neurons. J Neurosci.

[B9] Vaglio P, Lamesch P, Reboul J, Rual JF, Martinez M, Hill D, Vidal M (2003). WorfDB: the Caenorhabditis elegans ORFeome Database. Nucleic Acids Res.

[B10] Baugh LR, Hill AA, Slonim DK, Brown EL, Hunter CP (2003). Composition and dynamics of the Caenorhabditis elegans early embryonic transcriptome. Development.

[B11] Prasad BC, Ye B, Zackhary R, Schrader K, Seydoux G, Reed RR (1998). unc-3, a gene required for axonal guidance in Caenorhabditis elegans, encodes a member of the O/E family of transcription factors. Development.

[B12] Ogawa H, Harada S, Sassa T, Yamamoto H, Hosono R (1998). Functional properties of the unc-64 gene encoding a Caenorhabditis elegans syntaxin. J Biol Chem.

[B13] Saifee O, Wei L, Nonet ML (1998). The Caenorhabditis elegans unc-64 locus encodes a syntaxin that interacts genetically with synaptobrevin. Mol Biol Cell.

[B14] Hallam S, Singer E, Waring D, Jin Y (2000). The C. elegans NeuroD homolog cnd-1 functions in multiple aspects of motor neuron fate specification. Development.

[B15] Hedgecock EM, Culotti JG, Hall DH (1990). The unc-5, unc-6, and unc-40, guide circumferential migrations of pioneer axons and mesodermal cells on the nematode epidermis. Neuron.

[B16] Hobert O, Moerman DG, Clark KA, Beckerle MC, Ruvkun G (1999). A conserved LIM protein that affects muscular adherens junction integrity and mechanosensory function in Caenorhabditis elegans. J Cell Biol.

[B17] de la Cruz IP, Levin JZ, Cummins C, Anderson P, Horvitz HR (2003). sup-9, sup-10, and unc-93 may encode components of a two-pore K+ channel that coordinates muscle contraction in Caenorhabditis elegans. J Neurosci.

[B18] Nonet ML, Saifee O, Zhao H, Rand JB, Wei L (1998). Synaptic transmission deficits in Caenorhabditis elegans synaptobrevin mutants. Journal of Neuroscience.

[B19] Weimer RM, Jorgensen EM (2003). Controversies in synaptic vesicle exocytosis. J Cell Sci.

[B20] Eastman C, Horvitz HR, Jin Y (1999). Coordinated transcriptional regulation of the unc-25 glutamic acid decarboxylase and the unc-47 GABA vesicular transporter by the Caenorhabditis elegans UNC-30 homeodomain protein. J Neurosci.

[B21] McGhee JD, Krause M, D. A. Riddle TBBJMJRP (1997). Transcription Factors and Transcriptional Regulation. C elegans II.

[B22] Araujo SJ, Tear G (2003). Axon guidance mechanisms and molecules: lessons from invertebrates. Nat Rev Neurosci.

[B23] Wadsworth WG, Bhatt H, Hedgecock EM (1996). Neuroglia and pioneer axons express UNC-6 to provide global and local netrin cues for guiding migrations in Caenorhabditis elegans. Neuron.

[B24] Chang C, Yu TW, Bargmann CI, Tessier-Lavigne M (2004). Inhibition of netrin-mediated axon attraction by a receptor protein tyrosine phosphatase. Science.

[B25] Wills Z, Bateman J, Korey CA, Comer A, Van Vactor D (1999). The tyrosine kinase Abl and its substrate enabled collaborate with the receptor phosphatase Dlar to control motor axon guidance. Neuron.

[B26] Gitai Z, Yu TW, Lundquist EA, Tessier-Lavigne M, Bargmann CI (2003). The netrin receptor UNC-40/DCC stimulates axon attraction and outgrowth through enabled and, in parallel, Rac and UNC-115/AbLIM. Neuron.

[B27] Struckhoff EC, Lundquist EA (2003). The actin-binding protein UNC-115 is an effector of Rac signaling during axon pathfinding in C. elegans. Development.

[B28] Otsuka AJ, Boontrakulpoontawee P, Rebeiz N, Domanus M, Otsuka D, Velamparampil N, Chan S, Vande Wyngaerde M, Campagna S, Cox A (2002). Novel UNC-44 AO13 ankyrin is required for axonal guidance in C. elegans, contains six highly repetitive STEP blocks separated by seven potential transmembrane domains, and is localized to neuronal processes and the periphery of neural cell bodies. J Neurobiol.

[B29] Bloom L, Horvitz HR (1997). The Caenorhabditis elegans gene unc-76 and its human homologs define a new gene family involved in axonal outgrowth and fasciculation. Proc Natl Acad Sci U S A.

[B30] McIntire SL, Garriga G, White J, Jacobson D, Horvitz HR (1992). Genes necessary for directed axonal elongation or fasciculation in C. elegans. Neuron.

[B31] Ogura K, Shirakawa M, Barnes TM, Hekimi S, Ohshima Y (1997). The UNC-14 protein required for axonal elongation and guidance in Caenorhabditis elegans interacts with the serine/threonine kinase UNC-51. Genes Dev.

[B32] Lai T, Garriga G (2004). The conserved kinase UNC-51 acts with VAB-8 and UNC-14 to regulate axon outgrowth in C. elegans. Development.

[B33] Yoshikawa S, McKinnon RD, Kokel M, Thomas JB (2003). Wnt-mediated axon guidance via the Drosophila Derailed receptor. Nature.

[B34] Hirabayashi Y, Itoh Y, Tabata H, Nakajima K, Akiyama T, Masuyama N, Gotoh Y (2004). The Wnt/beta-catenin pathway directs neuronal differentiation of cortical neural precursor cells. Development.

[B35] Korswagen HC (2002). Canonical and non-canonical Wnt signaling pathways in Caenorhabditis elegans: variations on a common signaling theme. Bioessays.

[B36] Packard M, Koo ES, Gorczyca M, Sharpe J, Cumberledge S, Budnik V (2002). The Drosophila Wnt, wingless, provides an essential signal for pre- and postsynaptic differentiation. Cell.

[B37] Whangbo J, Kenyon C (1999). A Wnt signaling system that specifies two patterns of cell migration in C. elegans. Mol Cell.

[B38] Lyuksyutova AI, Lu CC, Milanesio N, King LA, Guo N, Wang Y, Nathans J, Tessier-Lavigne M, Zou Y (2003). Anterior-posterior guidance of commissural axons by Wnt-frizzled signaling. Science.

[B39] Jones AK, Sattelle DB (2004). Functional genomics of the nicotinic acetylcholine receptor gene family of the nematode, Caenorhabditis elegans. Bioessays.

[B40] Culetto E, Baylis HA, Richmond JE, Jones AK, Fleming JT, Squire MD, Lewis JA, Sattelle DB (2004). The caenorhabditis elegans unc-63 gene encodes a levamisole-sensitive nicotinic acetylcholine receptor alpha subunit. J Biol Chem.

[B41] Fleming JT, Squire MD, Barnes TM, Tornoe C, Matsuda K, Ahnn J, Fire A, Sulston JE, Barnard EA, Sattelle DB, Lewis JA (1997). Caenorhabditis elegans levamisole resistance genes lev-1, unc-29, and unc-38 encode functional nicotinic acetylcholine receptor subunits. J Neurosci.

[B42] Towers EB, Richmond JE, Sattelle DB (2005). The Caenorhabditis elegans lev-8 gene encodes a novel type of nicotinic acetylcholine receptor a subunit. J Neurochem.

[B43] Schafer WR (2002). Genetic analysis of nicotinic signaling in worms and flies. J Neurobiol.

[B44] Jones S, Sudweeks S, Yakel JL (1999). Nicotinic receptors in the brain: correlating physiology with function. Trends Neurosci.

[B45] Kim J, Poole DS, Waggoner LE, Kempf A, Ramirez DS, Treschow PA, Schafer WR (2001). Genes affecting the activity of nicotinic receptors involved in Caenorhabditis elegans egg-laying behavior. Genetics.

[B46] Halevi S, Yassin L, Eshel M, Sala F, Sala S, Criado M, Treinin M (2003). Conservation within the RIC-3 gene family. Effectors of mammalian nicotinic acetylcholine receptor expression. J Biol Chem.

[B47] Gally C, Eimer S, Richmond JE, Bessereau JL (2004). A transmembrane protein required for acetylcholine receptor clustering in Caenorhabditis elegans. Nature.

[B48] Brockie PJ, Madsen DM, Zheng Y, Mellem J, Maricq AV (2001). Differential expression of glutamate receptor subunits in the nervous system of Caenorhabditis elegans and their regulation by the homeodomain protein UNC-42. J Neurosci.

[B49] Lynch JW (2004). Molecular structure and function of the glycine receptor chloride channel. Physiol Rev.

[B50] Gomeza J, Ohno K, Hulsmann S, Armsen W, Eulenburg V, Richter DW, Laube B, Betz H (2003). Deletion of the mouse glycine transporter 2 results in a hyperekplexia phenotype and postnatal lethality. Neuron.

[B51] McIntire SL, Reimer RJ, Schuske K, Edwards RH, Jorgensen EM (1997). Identification and characterization of the vesicular GABA transporter. Nature.

[B52] Gomeza J, Hulsmann S, Ohno K, Eulenburg V, Szoke K, Richter D, Betz H (2003). Inactivation of the glycine transporter 1 gene discloses vital role of glial glycine uptake in glycinergic inhibition. Neuron.

[B53] Miller KG, Emerson MD, Rand JB (1999). Goalpha and diacylglycerol kinase negatively regulate the Gqalpha pathway in C. elegans. Neuron.

[B54] Nurrish S, Segalat L, Kaplan JM (1999). Serotonin inhibition of synaptic transmission: Galpha(0) decreases the abundance of UNC-13 at release sites. Neuron.

[B55] Chase DL, Pepper JS, Koelle MR (2004). Mechanism of extrasynaptic dopamine signaling in Caenorhabditis elegans. Nat Neurosci.

[B56] Lackner MR, Nurrish SJ, Kaplan JM (1999). Facilitation of synaptic transmission by EGL-30 Gqalpha and EGL-8 PLCbeta: DAG binding to UNC-13 is required to stimulate acetylcholine release. Neuron.

[B57] Steger KA, Avery L (2004). The GAR-3 muscarinic receptor cooperates with calcium signals to regulate muscle contraction in the Caenorhabditis elegans pharynx. Genetics.

[B58] Bany IA, Dong MQ, Koelle MR (2003). Genetic and cellular basis for acetylcholine inhibition of Caenorhabditis elegans egg-laying behavior. J Neurosci.

[B59] Schade MA, Reynolds NK, Dollins CM, Miller KG (2004). Mutations that Rescue the Paralysis of C. elegans ric-8 (Synembryn) Mutants Activate the G{alpha}s Pathway and Define a Third Major Branch of the Synaptic Signaling Network. Genetics.

[B60] Reynolds NK, Schade MA, Miller K (2005). Convergent, RIC-8 Dependent G{alpha} Signaling Pathways in the C. elegans Synaptic Signaling Network. Genetics.

[B61] Jansen G, Thijssen KL, Werner P, van der Horst M, Hazendonk E, Plasterk RH (1999). The complete family of genes encoding G proteins of Caenorhabditis elegans. Nat Genet.

[B62] Wilkie TM (1999). G proteins, chemosensory perception, and the C. elegans genome project: An attractive story. Bioessays.

[B63] Moresco JJ, Koelle MR (2004). Activation of EGL-47, a Galphao-coupled receptor, inhibits function of hermaphrodite-specific motor neurons to regulate Caenorhabditis elegans egg-laying behavior. J Neurosci.

[B64] Dong MQ, Chase D, Patikoglou GA, Koelle MR (2000). Multiple RGS proteins alter neural G protein signaling to allow C. elegans to rapidly change behavior when fed. Genes Dev.

[B65] Keating CD, Kriek N, Daniels M, Ashcroft NR, Hopper NA, Siney EJ, Holden-Dye L, Burke JF (2003). Whole-genome analysis of 60 G protein-coupled receptors in Caenorhabditis elegans by gene knockout with RNAi. Curr Biol.

[B66] Li C, Kim K, Nelson LS (1999). FMRFamide-related neuropeptide gene family in Caenorhabditis elegans. Brain Res.

[B67] Kim K, Li C (2004). Expression and regulation of an FMRFamide-related neuropeptide gene family in Caenorhabditis elegans. J Comp Neurol.

[B68] Nelson LS, Rosoff ML, Li C (1998). Disruption of a neuropeptide gene, flp-1, causes multiple behavioral defects in Caenorhabditis elegans. Science.

[B69] Rogers C, Reale V, Kim K, Chatwin H, Li C, Evans P, de Bono M (2003). Inhibition of Caenorhabditis elegans social feeding by FMRFamide-related peptide activation of NPR-1. Nat Neurosci.

[B70] Rogers CM, Franks CJ, Walker RJ, Burke JF, Holden-Dye L (2001). Regulation of the pharynx of Caenorhabditis elegans by 5-HT, octopamine, and FMRFamide-like neuropeptides. J Neurobiol.

[B71] Pierce SB, Costa M, Wisotzkey R, Devadhar S, Homburger SA, Buchman AR, Ferguson KC, Heller J, Platt DM, Pasquinelli AA, Liu LX, Doberstein SK, Ruvkun G (2001). Regulation of DAF-2 receptor signaling by human insulin and ins-1, a member of the unusually large and diverse C. elegans insulin gene family. Genes Dev.

[B72] Nathoo AN, Moeller RA, Westlund BA, Hart AC (2001). Identification of neuropeptide-like protein gene families in Caenorhabditiselegans and other species. Proc Natl Acad Sci U S A.

[B73] Jacob TC, Kaplan JM (2003). The EGL-21 carboxypeptidase E facilitates acetylcholine release at Caenorhabditis elegans neuromuscular junctions. J Neurosci.

[B74] Kass J, Jacob TC, Kim P, Kaplan JM (2001). The EGL-3 proprotein convertase regulates mechanosensory responses of Caenorhabditis elegans. J Neurosci.

[B75] Zahn TR, Angleson JK, MacMorris MA, Domke E, Hutton JF, Schwartz C, Hutton JC (2004). Dense core vesicle dynamics in Caenorhabditis elegans neurons and the role of kinesin UNC-104. Traffic.

[B76] Spitzenberger F, Pietropaolo S, Verkade P, Habermann B, Lacas-Gervais S, Mziaut H, Pietropaolo M, Solimena M (2003). Islet cell autoantigen of 69 kDa is an arfaptin-related protein associated with the Golgi complex of insulinoma INS-1 cells. J Biol Chem.

[B77] Pilon M, Peng XR, Spence AM, Plasterk RH, Dosch HM (2000). The diabetes autoantigen ICA69 and its Caenorhabditis elegans homologue, ric-19, are conserved regulators of neuroendocrine secretion. Mol Biol Cell.

[B78] Cai T, Fukushige T, Notkins AL, Krause M (2004). Insulinoma-Associated Protein IA-2, a Vesicle Transmembrane Protein, Genetically Interacts with UNC-31/CAPS and Affects Neurosecretion in Caenorhabditis elegans. J Neurosci.

[B79] Richmond JE, Broadie KS (2002). The synaptic vesicle cycle: exocytosis and endocytosis in Drosophila and C. elegans. Curr Opin Neurobiol.

[B80] Davis RE, Stretton AO (2001). Structure-activity relationships of 18 endogenous neuropeptides on the motor nervous system of the nematode Ascaris suum. Peptides.

[B81] Doi M, Iwasaki K (2002). Regulation of retrograde signaling at neuromuscular junctions by the novel C2 domain protein AEX-1. Neuron.

[B82] Mello CC, Draper BW, Krause M, Weintraub H, Priess JR (1992). The pie-1 and mex-1 genes and maternal control of blastomere identity in early C. elegans embryos. Cell.

[B83] Portman DS, Emmons SW (2004). Identification of C. elegans sensory ray genes using whole-genome expression profiling. Dev Biol.

[B84] Zhang Y, Ma C, Delohery T, Nasipak B, Foat BC, Bounoutas A, Bussemaker HJ, Kim SK, Chalfie M (2002). Identification of genes expressed in C. elegans touch receptor neurons. Nature.

[B85] McKay SJ, Johnsen R, Khattra J, Asano J, Baillie DL, Chan S, Dube N, Fang L, Goszczynski B, Ha E, Halfnight E, Hollebakken R, Huang P, Hung K, Jensen V, Jones SJ, Kai H, Li D, Mah A, Marra M, McGhee J, Newbury R, Pouzyrev A, Riddle DL, Sonnhammer E, Tian H, Tu D, Tyson JR, Vatcher G, Warner A, Wong K, Zhao Z, Moerman DG (2003). Gene expression profiling of cells, tissues, and developmental stages of the nematode C. elegans. Cold Spring Harb Symp Quant Biol.

[B86] Colosimo ME, Brown A, Mukhopadhyay S, Gabel C, Lanjuin AE, Samuel AD, Sengupta P (2004). Identification of thermosensory and olfactory neuron-specific genes via expression profiling of single neuron types. Curr Biol.

[B87] Roy PJ, Stuart JM, Lund J, Kim SK (2002). Chromosomal clustering of muscle-expressed genes in Caenorhabditis elegans. Nature.

[B88] Kunitomo H, Uesugi H, Kohara Y, Iino Y (2005). Identification of ciliated sensory neuron-expressed genes in Caenorhabditis elegans using targeted pull-down of poly(A) tails. Genome Biol.

[B89] Sulston JE (1983). Neuronal cell lineages in the nematode Caenorhabditis elegans. Cold Spring Harbor Symposia on Quantitative Biology.

[B90] Geary TG, Kubiak TM (2005). Neuropeptide G-protein-coupled receptors, their cognate ligands and behavior in Caenorhabditis elegans. Trends Pharmacol Sci.

[B91] Grevengoed EE, Fox DT, Gates J, Peifer M (2003). Balancing different types of actin polymerization at distinct sites: roles for Abelson kinase and Enabled. J Cell Biol.

[B92] White JG, Southgate E, Thomson JN (1992). Mutations in the Caenorhabditis elegans unc-4 gene alter the synaptic input to ventral cord motor neurons. Nature.

[B93] Winnier AR, Meir JY, Ross JM, Tavernarakis N, Driscoll M, Ishihara T, Katsura I, Miller DM (1999). UNC-4/UNC-37-dependent repression of motor neuron-specific genes controls synaptic choice in Caenorhabditis elegans. Genes Dev.

[B94] Ao W, Gaudet J, Kent WJ, Muttumu S, Mango SE (2004). Environmentally induced foregut remodeling by PHA-4/FoxA and DAF-12/NHR. Science.

[B95] Hill AA, Brown EL, Whitley MZ, Tucker-Kellogg G, Hunter CP, Slonim DK (2001). Evaluation of normalization procedures for oligonucleotide array data based on spiked cRNA controls. Genome Biol.

[B96] Irizarry RA, Bolstad BM, Collin F, Cope LM, Hobbs B, Speed TP (2003). Summaries of Affymetrix GeneChip probe level data. Nucleic Acids Res.

[B97] Irizarry RA, Hobbs B, Collin F, Beazer-Barclay YD, Antonellis KJ, Scherf U, Speed TP (2003). Exploration, normalization, and summaries of high density oligonucleotide array probe level data. Biostatistics.

[B98] Storey JD, Tibshirani R (2003). Statistical significance for genomewide studies. Proc Natl Acad Sci U S A.

[B99] Tusher VG, Tibshirani R, Chu G (2001). Significance analysis of microarrays applied to the ionizing radiation response. Proc Natl Acad Sci U S A.

[B100] Brenner S (1974). The genetics of Caenorhabditis elegans. Genetics.

[B101] Mello C, Fire A (1995). DNA transformation. Methods in Cell Biology.

[B102] Dupuy D, Li QR, Deplancke B, Boxem M, Hao T, Lamesch P, Sequerra R, Bosak S, Doucette-Stamm L, Hope IA, Hill DE, Walhout AJ, Vidal M (2004). A First Version of the Caenorhabditis elegans Promoterome. Genome Res.

[B103] Praitis V, Casey E, Collar D, Austin J (2001). Creation of low-copy integrated transgenic lines in Caenorhabditis elegans. Genetics.

[B104] Pflugrad A, Meir JY, Barnes TM, Miller DM (1997). The Groucho-like transcription factor UNC-37 functions with the neural specificity gene unc-4 to govern motor neuron identity in C. elegans. Development.

[B105] Li C, Nelson LS, Kim K, Nathoo A, Hart AC (1999). Neuropeptide gene families in the nematode Caenorhabditis elegans. Ann N Y Acad Sci.

[B106] Salkoff L, Butler A, Fawcett G, Kunkel M, McArdle C, Paz-y-Mino G, Nonet M, Walton N, Wang ZW, Yuan A, Wei A (2001). Evolution tunes the excitability of individual neurons. Neuroscience.

[B107] Sym M, Robinson N, Kenyon C (1999). MIG-13 positions migrating cells along the anteroposterior body axis of C. elegans. Cell.

