# Supplementary material for: A gene expression fingerprint of C. elegans embryonic motor neurons
Source: BMC Genomics. 2005 Mar 21;6:42. doi: 10.1186/1471-2164-6-42 (PMC1079822; doi:10.1186/1471-2164-6-42)
Supplement: Additional File 16 — Gene families represented in unc-4::GFP neurons. A comprehensive description of neuronal transcripts organized according to gene family. [file 1471-2164-6-42-S16.doc]

Additional File 16

Descriptions of additional gene families with potential roles in motor neuron differentiation and function

Genes written in bold type are enriched in *unc-4*::GFP motor neurons. All other transcripts listed here are detected as EGs unless otherwise indicated.

**Transcription factors.**
In the embryo, the **UNC-4** transcription factor is exclusively expressed in I5, SAB and DA motor neurons (Fig 2D,5A) [1]. It is therefore not surprising that ***unc-4*** is one of the most highly enriched transcripts in our data set (See table below, Additional File 9). **UNC-4** and its homologs in other species are members of the Prd-like class of homeodomain proteins and are likely to function as transcriptional repressors [2, 3]. In *C. elegans*, **UNC-4** physically interacts with UNC-37, a Groucho-like transcriptional co-repressor protein [3, 4]. The ubiquitous expression of UNC-37 *in vivo* is consistent with our finding that the *unc-37* transcript is an EG but is not enriched in the *unc-4*::GFP motor neuron data set. **UNC-4** activity is required for normal SAB axonal outgrowth and for maintaining wildtype levels of neurotransmitter vesicles in SAB and DA motor neurons [5]. Downstream **UNC-4** targets that regulate these events are unknown.

A member of the highly conserved OLF-1/EBF family of transcription factors, **UNC-3** is expressed in a subset of cholinergic ventral cord motor neurons including the DAs and in the ASI sensory neuron. ***unc-3*** mutants show defects in motor neuron axon outgrowth and in ASI function [6]. These data are consistent with the elevated level of ***unc-3*** transcript in the *unc-4*::GFP motor neuron data set (See table below, Additional File 9). Vertebrate OLF-1/EBF proteins also control neuronal differentiation and axonal morphology in the CNS [7].

**MAB-9** is a member of the T-box family of transcription factors [8]. Of the 20 T-box transcription factor genes in the *C. elegans* genome, only ***mab-9*** is enriched in UNC-4 motor neurons. We used a ***mab-9***::GFP reporter to confirm expression in DA motor neurons (Table 1). **MAB-9** also functions in the GABAergic motor neurons to control axonal outgrowth and morphology [9] (Pocock and Woollard, Oxford, personal communication). The role of **MAB-9** in motor neuron differentiation may be conserved as the vertebrate homolog, TBX20, is highly expressed in hindbrain and spinal motor neurons [10, 11].

***ces-1*** encodes a Snail-like transcription factor that functions in the NSM serotonergic motor neurons to block expression of the apoptotic factor, EGL-1 [12]. The most closely related protein in *Drosophila*, *Scratch*, is widely expressed in neuroblasts whereas the vertebrate homolog is restricted to subsets of postmitotic neurons in the brain and spinal cord [13]. A function for **CES-1** in *C. elegans* ventral cord motor neurons has not been previously reported.

The *C. elegans* genome includes 284 genes with significant similarity to nuclear hormone receptors (NRs) [14]. Six NR genes (***nhr-3, -95, -104, -116*, F41B5.9, C29G2.5**) are enriched in UNC-4 motor neurons. To date, no functions have been assigned to these genes. An additional 43 NRs are present but not enriched in this data set (Additional File 14). Among these NRs is *daf-12*, a presumptive steroid hormone receptor gene that is widely expressed in most cells to control dauer formation and adult longevity [15]. In the vertebrate spinal cord, retinoic acid is a potent inducer of specific neuronal fates [16]. The strong similarity of ***nhr-3*** (BLAST = e^-28^) to the mammalian retinoic acid NR could be indicative of an ancient role for steroid hormone signaling in motor neuron differentiation. Significantly, *fax-1*, *unc-55* and *odr-7* regulate differentiation of specific neurons (i.e. AVK, VD, AWA respectively) [17-19] and are not detected in *unc-4*::GFP motor neurons (See table below, Additional File 8).

Five additional transcription factor mRNAs with potential functions in UNC-4 motor neuron differentiation are enriched: the GATA factor ***elt-1***, **C27C12.6** [DM (Doublesex-mab-3) Zn finger DNA binding domain]; **B0336.13** [TFIIA, a component of the basal transcription complex]; **F44E2.6** [pillin-like transcription factor]; **T08H4.3** [FLI-1 (Friend Leukemia integration 1 transcription factor) ETS domain]. Functions for these transcription factors in UNC-4 motor neurons have not been described.

# Cell Adhesion Molecules

Cell adhesion molecules define the architecture and connectivity of the nervous system. The cadherin and Ig superfamily proteins are of particular interest because of their key roles in axon guidance and synaptic specificity.

The *C. elegans* genome encodes 80 Immunoglobulin (Ig) domain proteins [20-22]. Transcripts for five Ig superfamily Cell Adhesion Molecules (IgCAMs), **SYG-1, SAX-7, C33F10.5, C53A5.13** and **RIG-3** are enriched in UNC-4 motor neurons. All of these proteins display extracellular protein interaction domains composed of Ig repeats (**SYG-1, RIG-3**) or tandem arrays of Ig and fibronectin repeats (**SAX-7**, **C33F10.5**, **C53A5.13**). **SYG-1**, **SAX-7** and **C53A5.13** all contain single-pass transmembrane domains while **C33F10.5** and **RIG-3** have consensus GPI attachment sites [23]. Intercellular contact of **SYG-1** with the related Ig domain protein, SYG-2 (not detected), marks the location of a specific synapse in the egg laying circuit [24, 25]. We have confirmed that ***syg-1***::GFP is expressed in DA motor neurons (Table 1) and speculate that it may specify presynaptic sites in these cells. ***sax-7*** encodes the nematode ortholog of L1CAM/neuroglian a key mediator of axonal pathfinding and morphology in mammals and in *Drosophila* [26, 27]. **SAX-7** is widely expressed and localizes to intercellular contacts during epithelial and gonad morphogenesis in *C. elegans*. **SAX-7** function at these sites may depend on interactions with the ankyrin like cytoskeletal protein, **UNC-44**, which is also enriched in *unc-4*::GFP motor neurons (See table below, Additional File 9) [28]. **SAX-7** may also have a role in maintaining the structure of the *C. elegans* nervous system as the adult nerve ring is disorganized in ***sax-7*** mutants [29]. In flies and mammals, L1CAM/neuroglian forms a complex with another IgCAM, Contactin, at septate junctions associated with neuron ensheathment sites [30, 31]. Although nematode neurons are not insulated, the contactin-like molecule, **C33F10.5**, is enriched in UNC-4 motor neurons and therefore may have engaged L1CAM/Neuroglian in a primordial function that precedes the evolution of myelination strategies. **RIG-3** is related to klingon, a GPI-anchored protein [20] that mediates homophilic interactions that define fly photoreceptor development [32]. Finally, **C53A5.13** shows weak similarity to the L1CAM family and therefore may encode a nematode-specific Ig protein. The selective enrichment of this subset IgCAMS points to specific roles for these adhesion proteins in cell-cell interactions that govern the differentiation or function of UNC-4 motor neurons.

Cadherins are single pass transmembrane proteins with large ectodomains that mediate Ca^++^ dependent homotypic interactions crucial to tissue morphogenesis [33]. In the nervous system, cadherins are concentrated at synapses and may facilitate target recognition [34, 35]. *C. elegans* contains 15 genes encoding proteins with cadherin-like extracellular domains [33]. The single classical cadherin gene, *hmr-1*, is alternatively spliced to generate two transcripts, *hmr-1a* and *hmr-1b*. HMR-1a is important for epithelial morphogenesis whereas HMR-1b is necessary for axonal pathfinding, especially in D-class motor neurons [36]. *hmr-1* is not enriched, but is detected as an EG. One cadherin transcript, ***cdh-11***, is enriched in UNC-4 neurons and encodes the nematode ortholog of calsyntenin, a postsynaptically localized protein of unknown function that is highly expressed in the mammalian brain [37]. A role for **CDH-11** in the *C. elegans* nervous system has not been described. Five other cadherin transcripts (*cdh-1,-4, -5, -6, -7*) are EGs.

Transcripts for two muscle proteins, **UNC-22**, a sarcomeric protein with multiple Ig and FN repeats [38], and **UNC-112**, a muscle cell attachment site component with pleckstrin homology domains [39], and two additional proteins **AJM-1**, a novel coiled-coil constituent of apical junctions in epithelial cells [40], and **T19D12.6**, a conserved protein with three lamininG domains, are also enriched. Functional tests are required to define the potential roles of these genes in UNC-4 motor neurons.

**Synapse-Associated Proteins**

Genetic approaches in *C. elegans* have identified genes with key roles in synapse formation. In a screen to find GABAergic Synapse Defective (*syd*) genes, Yishi Jin's lab identified ***syd-1*** (rho-GAP activity with PDZ domain) and ***syd-2*** (α-Liprin, LAR interacting protein) [41, 42]. Both are enriched in the *unc-4*::GFP dataset. **SYD-1** likely acts through its rho-GAP domain to specify axon identity; **SYD-2** is necessary for active zone formation. In mammals, **SYD-2** homologs interact with LAR (Leukocyte common Antigen-Related protein), a receptor protein-tyrosine phophastase (RPTP) [43]. LAR activity defines the size, number and target projections of basal forebrain cholinergic neurons in the mouse [44]. In *Drosophila*, DLAR is important for axon guidance and target recognition in the visual system [45, 46]. ***ptp-3*** encodes the *C. elegans* homolog of LAR. ***ptp-3*** is required for epidermal enclosure in the embryo and is also highly expressed in the *C. elegans* nervous system [47]. The coincident enrichment of ***syd-2*** and ***ptp-3*** in UNC-4 motor neurons may be indicative of important roles in process outgrowth or target recognition in the motor neuron circuit. A second RPTP (**K07F5.6**) is also enriched but its potential function is unknown.

**RPM-1** (Regulator of Presynaptic Morphology) is a large, highly conserved component of the perisynaptic region adjacent to the active zone. Synaptogenesis and axon outgrowth depend on **RPM-1** E3 ubiquitin ligase activity [48-50]. **RPM-1** is expressed in all neurons, thus validating its detection in the *unc-4*::GFP motor neuron data set. We also detected enrichment of a nearby gene, **F07B7.12***,* that is virtually identical to the ***rpm-1*** coding sequence and appears to have arisen as a recent, partial duplication of the ***rpm-1*** locus; **F07B7.12** lacks the **RPM-1** C-terminal E3 ubiquitin ligase domain and is not present in the *C. briggsae* genome (data not shown). The function of the **F07B7.12** protein is unknown.

Rapsyn (***rpy-1****)* is a post-synaptic component of the NMJ where it functions to cluster acetylcholine receptors (AChRs) [51]. Enrichment of the ***rpy-1*** transcript in UNC-4 motor neurons could be related to co-expression of multiple nicotinic acetylcholine receptors (nAChRs) in these cells (see Results).

***lin-2****, lin-7,* and ***lin-10*** encode PDZ domain components of a protein complex that localizes the LET-23/EGF receptor during vulval development [52]. An additional role for **LIN-10** in glutamate receptor trafficking has also been discovered [53]. Transcripts for two of these proteins are enriched (***lin-2*, *lin-10***) and one is an EG (*lin-7)*, thereby suggesting potential roles in receptor localization in UNC-4 motor neurons.

# Neurotransmitter Vesicular Release

Synaptic transmission is triggered by Ca++ dependent fusion of synaptic vesicles (SVs) with the presynaptic membrane to release neurotransmitters (NTs) into the synaptic cleft. Nascent SVs are generated in the soma for translocation to axonal termini where they are loaded with neurotransmitter. The arrival of an action potential stimulates exocytosis. SVs are regenerated by an endocytic process that recycles the SV membrane for reimportation of NTs in the presynaptic zone [54]. Most of the known constituents of the SV cycle are detected in the *unc-4*::GFP motor neuron data set.

### Synaptic Vesicle Trafficking

Synaptic vesicles are transported from cell soma to the axon terminal via motor-dependent mechanisms. The kinesin heavy chains *unc-104* and *unc-116* are required to mediate this process [55, 56] and both are detected as EGs; the kinesin light chain, ***klc-2*** is enriched. Recent reports show that UNC-116 and **KLC-2** form a functional kinesin-1 complex in neurons [57]. UNC-16, Jnk-kinase interacting protein (JIP), has been proposed to interact with **UNC-14** as a molecular tether between kinesin-1 and synaptic vesicles [56, 57]. UNC-16 also interacts with JNK *(****jnk-1***) as well as JNK-kinase (***jkk-1***) indicating that Jun-kinase signaling is critical for trafficking of synaptic proteins. Mutations in all of these components lead to mislocalization of synaptic vesicle components.

### Vesicle loading

*unc-4*::GFP motor neurons are excitatory and release the neurotransmitter acetylcholine. ***unc-17***, the vesicular acetylcholine transporter (VChAT) and the ACh synthetic enzyme, ***cha-1*** (choline acetyltransferase) are co-expressed in all cholinergic neurons [58]. Neurotransmitter loading depends on an electrochemical proton gradient established by the Vacuolar ATPase pump and various subunits of this complex (e.g. *unc-32*) [59] are detected as EGs. Nematode orthologs of SV2 (**ZK637.1**) and SVOP (**Y51A2D.18**), two members of the 12 TM domain transporter superfamily, are enriched. SV2 and SVOP functions are unknown but both are integral synaptic vesicle membrane proteins [60, 61].

### Vesicle exocytosis and Neurotransmitter release

SV exocytosis is achieved via a stepwise process of vesicle docking, maturation (priming) and fusion [62]. Transcripts for most of the genes known to encode proteins involved in these events are detected in the *unc-4*::GFP motor neuron data set. Strikingly, Synaptogyrin (***sng-1***), a regulator of exocytosis [59], is one of the most highly ranked genes (See table below, Additional File 9). Components of the SNARE complex that mediate SV association with the presynaptic membrane [63] are either enriched (**Y22F5A.3, SNAP-25**) or detected as EGs (K02D10.5, SNAP-25-like protein; *snb-1*, Synaptobrevin; *unc-64*, Syntaxin). Genes important for SV fusion, ***unc-18*** (Sec1p family), ***rab-3*** (GTPase associated with SV)***,*** and ***unc-1******0*** (RIM) are enriched in these cells, while a key regulator of the priming step, *unc-13* is detectable as an EG. As expected, ***snt-1*** (synaptotagmin) the calcium sensor that triggers NT release, is enriched [59]. Other enriched synaptic transmission genes include the SV protein Synapsin (***snn-1***), and the guanine nucleotide exchange factor for Rab3 (***aex-3***) [64, 65]. We note that the piccolo-like protein, **F45E4.3**, a proposed component of the vesicle priming complex that includes UNC-13 and RIM (**UNC-10**) [66], is enriched in UNC-4 motor neurons. Another enriched transcript with a potential role in vesicular release, **F54G2.1**, encodes the nematode ortholog of BAP3, an UNC-13-related gene that is highly expressed in the human brain [67, 68].

**Vesicle** **Endocytosis**

Clathrin-mediated endocytosis is the principle pathway for recycling synaptic vesicle membranes [54, 59, 63]. In the first step, clathrin adaptor proteins are recruited to the site of endocytosis by synaptic vesicle proteins. These interactions are required for the efficient recovery of both SV lipids and membrane proteins. For example, the AP180 clathrin adaptor protein, **UNC-11**, is specifically required for recycling synaptobrevin (SNT-1) [69]. Additional components of the clathrin adaptor complexes are either enriched (***apt-2, apt-4***, ***aps-2*)** or detected as EGs (*apt-1, apt-6,* *apt-7*, *dpy-23,* *tag-11)*. The accessory proteins and lipid modifying enzymes UNC-26 (Synaptojanin) and **UNC-57** (endophilin) are required for recruitment of the AP2 adaptor complex by Synaptotagmin (**SNT-1**) [70, 71]. Separation of the endocytic vesicle from the plasma membrane or vesicle fission is driven by the GTPase dynamin (*dyn-1*). Other genes that facilitate vesicle endocytosis are either enriched [***apt-10*** (Stoned B)] or EGs [F58G6.1 (amphiphysin), and *ehs-1* (EPS15)]. The clathrin coat is removed in the final stage of endocytosis to release the nascent SV. The heat shock protein Hsc70 (*hsp-1*) and the DnaJ protein Auxilin (*dnj-25*) are critical for this process [63] and both are EGs in the *unc-4*::GFP motor neuron data set. Finally, the transcript for **RME-8**, a novel J-domain protein required for endocytosis in coelomocytes and in the somatic gonad [72] is enriched; a function for ***rme-8*** in the nervous system has not been previously reported.

### TGF-β Signaling

Transcripts for three of the four known BMP-4/TGF-β peptides in *C. elegans* [81] are either enriched (***tig-2***) or detected as EGs (*dbl-1*, *unc-129*). Expression of *dbl-1* and *unc-129* in DA motor neurons has been previously described [82, 83] and we have used a GFP reporter to confirm ***tig-2*** expression in these cells (Fig 5). It is interesting to note that *dbl-1*, *unc-129*, and ***tig-2*** are also expressed in body wall muscles. In *Drosophila*, formation of a normal neuromuscular synapse depends on reciprocal BMP-4/TGF-β signaling between motor neurons and target muscles [84]. A comparable role for BMP/TGF-β signaling at the nematode neuromuscular synapse has not been detected in BMP signaling mutants. Perhaps, this outcome is a result of redundant BMP/TGF-β signals.

### Serpentine Receptors

The members of the chemosensory receptor gene family (also known as the serpentine receptors [SR]) constitute ~6% (~1300 genes) of the *C. elegans* genome. Many of these genes have arisen from recent gene duplications, and almost 1/3 are predicted to be pseudogenes [85, 86]. In mammals and in *C. elegans*, G-protein coupled receptors are required for odorant discrimination [87]. *C. elegans* can detect hundreds of different compounds which suggests that a significant fraction of the SR receptors are utilized. Due to the large number of receptors (>500), and the relatively small number of chemosensory neurons (20-30) in *C. elegans*, each neuron is likely to express 40-50 different receptors [85, 87, 88]. Transcripts for 18 SRs are enriched and 96 transcripts are detected as EGs. Because *unc-4*::GFP neurons are components of the motor circuit, it seems unlikely that SRs are functioning as odorant receptors in these cells. It will be interesting to determine if members of the SR family are widely expressed in other classes of *C. elegans* neurons.

**Calcium Channels**

Synaptic transmission is triggered by calcium influx via voltage gated calcium channels [59]. Calcium channels are composed of distinct subunits: α1, α2δ, β, γ. Electrophysiological studies have identified distinct calcium currents; L-, N-, P-, Q-, R-, and T-type. These are largely defined by the specific type of α1 subunit incorporated into the channel [89]. The ***unc-2*** gene encodes an α1 subunit of the N-/P-/Q-class and is highly expressed in motor neurons. Pharmacologic studies indicate that the impaired movement shown by ***unc-2*** mutants is a consequence of presynaptic defects in neurotransmitter release at the neuromuscular junction [90]. Our finding that ***unc-2*** is enriched in the *unc-4*::GFP motor neuron data set is consistent with this model. *unc-36* (EG) encodes an α2δ subunit and has been proposed to co-assemble with **UNC-2** on the basis of similar mutant phenotypes [91]. Another α2δ type subunit, **T24F1.6**, is enriched but has not been genetically characterized. A β subunit encoding transcript (*ccb-1*) is an EG. (γ subunit genes have not been identified in *C. elegans* [92]).

The *C. elegans* genome includes two α1-like subunit genes, ***nca-1*** and *nca-2*, that encode large so-called 4-domain calcium channel subunits (K. Hamming, C. Thacker, T. Snutch, personal communication); ***nca-1*** is enriched in UNC-4 motor neurons and a GFP reporter confirms expression in DA and in other cholinergic ventral cord motor neurons (Table I). *nca-2* is not represented on the Affymetrix Chip and therefore is not detected (data not shown). ***nca-1*** and *nca-2* are highly conserved with apparent human orthologs but their functions are unknown.

Other classes of calcium transporters with well-established roles in neuron excitability are enriched in UNC-4 motor neurons. These include channels that release calcium from internal stores (***unc-68***, ryanodine receptor) as well as transporters that remove calcium from the cytoplasm (***mca-3***, PMCA1-type calcium-transporting ATPase and ***ncx-4***, Ca^2+^/Na^2+^ exchanger) [93, 94]. Although **UNC-68** expression in *C. elegans* neurons has not been previously reported, mammalian ryanodine receptors are highly expressed in the CNS where they co-localize with L-type voltage gated calcium channels to mediate excitation-coupled calcium release from the endoplasmic reticulum [95]. Lastly, ***trp-1,*** a TRPC (TRPCanonical) calcium/cation channel of the TRP (Transient Receptor Potential) superfamily, is enriched in the *unc-4*::GFP motor neuron data set, and we have confirmed its expression in UNC-4 motor neurons (Table 1). In other cell types, TRP channels are gated by environmental stimuli (e.g., temperature or mechanical disturbance) but potential functions of TRPC channel in these motor neurons are unknown [96].

### Calcium ion binding

The calcium-binding protein, calmodulin regulates calcium-dependent signaling pathways that control multiple aspects of neuronal function including synaptic activity and gene expression [97]. *C. elegans* encodes a single, highly conserved calmodulin ortholog, ***cmd-1*** and four calmodulin-like genes [98]. Transcripts for ***cmd-1*** and two of the calmodulin-like genes (**Y73B3A.12, F12A10.5**) are enriched in UNC-4 motor neurons. CAM kinase activity is regulated by calmodulin [99] and all of the canonical CAMK components, *unc-43* (CAMKII), *cmk-1* (CAMKI), and *ckk-1* (CAMKK), are detected as EGs. Another downstream effector of calmodulin is calcineurin, a heterodimeric serine/threonine phosphatase; the catalytic A subunit (*tax-6*) is an EG and the Regulatory B subunit (***cnb-1***) is enriched in the *unc-4*::GFP motor neuron data set. These data are consistent with genetic evidence showing that *unc-43*/CAMKII and calcineurin are antagonistic regulators of G-protein dependent locomotion in *C. elegans* [100, 101]. The transcript for a calcineurin regulatory protein, *rcn-1*, is detected as an EG in these cells [100]. Thus, key conserved components of calmodulin-dependent signaling pathways are expressed in *unc-4*::GFP motor neurons.

Transcripts for other notable calcium binding proteins enriched in UNC-4 motor neurons include: **R08D7.5**, centrin/caltractin, a member of the calmodulin superfamily and component of the microtubule organizing center [102]; ***pef-1***, serine/threonine protein phosphatase, an ortholog of PPEF/rdgC (retinal degenration gene C) and proposed regulator of G-protein activity [103]; ***spc-1***, alpha-spectrin, an actin binding and scaffolding protein [104]; ***nex-1***, annexin, an actin and phospholipid binding protein [105].

# Potassium Channels

Potassium channels are subdivided into three main groups on the basis of the number of transmembrane domains within each subunit: 2 TM inward rectifiers, 4 TM (2 pore) or TWIKs, and 6 TM voltage gated channels [106]. TWIK channel encoding genes are the largest and most evolutionarily diverged group with ~40 members in the *C. elegans* genome. We find 8 members of the TWIK family enriched in UNC-4 motor neurons (***twk-6, -13, -29, -30, -40, -46, unc-58*, R05G9.28**). VNC motor neuron expression of ***twk-30*** and ***unc-58*** has been previously reported [107], and we have confirmed that ***twk-30***::GFP is expressed in DA motor neurons (Table I). TWIK channels are believed to set resting membrane potential and hence modulate cell excitability. A physiological requirement for multiple potentially redundant TWIK channels in these motor neurons is unclear although this arrangement may allow for “fine tuning” of the electrical responsiveness of these cells[107]. Two voltage gated K channels, F44A2.2 (Shab/K2v.2) and M60.5 (KQT) are detected as EGs.

### Innexins

Neurons are electrically coupled by gap junctions, intercellular channels that facilitate the movement of ions and small molecules between cells. Gap junctions are multimeric membrane pores assembled from protein subunits contributed by each cell [108]. The invertebrate gap junction is composed of innexins, which are structurally similar to vertebrate gap junction subunits, connexins. There are 25 innexin genes in *C. elegans* [109]. Only one of these, ***unc-9***, is enriched in the *unc-4*::GFP motor neuron data set. ***unc-9*** mutants show jerky, uncoordinated movements indicative of defective function in the motor circuit [108]. These mutant effects could arise from the disruption of gap junctions that electrically couple DA motor neurons with command interneurons and with other motor neurons [110]. Five additional innexin genes (*inx-1, -6, -7, -15, -22*) are detected as EGs, which could mean that UNC-4 motor neurons assemble more than one type of gap junction. It is surprising that the *unc-7* transcript is not detected in these cells as *unc-7* and ***unc-9*** mutants exhibit similar mutant phenotypes indicative of function in a common genetic pathway [108].

### DEG/ENaC and Stomatins

DEG/ENaC sodium channels are comprised of single TM domain subunits that are believed to gate cation transport in response to mechanical force [111]. There are 21 DEG/ENaC encoding genes in *C. elegans* [111]. The transcript for one of these, ***unc-8***, is enriched, and confirms previous reports of ***unc-8*** expression in VNC motor neurons. The **UNC-8** protein has been proposed to function as a “stretch receptor” in a feed-back loop to coordinate motor neuron excitability with muscle contraction [112]. DEG/ENaC activity in *C. elegans* touch neurons is modulated by interactions with Stomatin-like proteins (SLPs) [113]. **UNC-8** physically interacts with the SLP **UNC-1** [114]. Consistent with this finding, the ***unc-1*** transcript is enriched in the *unc-4*::GFP motor neuron data set. In addition, the novel SLP *unc-24*, which may also modulate ***unc-8*** activity in the DA motor neurons is an EG (J. Koh, and DMM, unpublished data). Finally, the SLP, ***sto-6***, is enriched, as predicted by GFP expression [115], but its function is unknown.

**References**

1. DM Miller, III, CJ Niemeyer: **Expression of the *unc-4* homeoprotein in *C. elegans* motor neurons specifies presynaptic input**. *Development* 1995, **121**:2877-2866.

2. DM Miller, MM Shen, CE Shamu, TR Burglin, G Ruvkun, ML Dubois, M Ghee, L Wilson: ***C. elegans* *unc-4* gene encodes a homeodomain protein that determines the pattern of synaptic input to specific motor neurons**. *Nature* 1992, **355**:841-5.

3. AR Winnier, JY Meir, JM Ross, N Tavernarakis, M Driscoll, T Ishihara, I Katsura, DM Miller, 3rd: **UNC-4/UNC-37-dependent repression of motor neuron-specific genes controls synaptic choice in *C. elegans***. *Genes Dev* 1999, **13**:2774-2786.

4. A Pflugrad, JY Meir, TM Barnes, DM Miller, 3rd: **The Groucho-like transcription factor UNC-37 functions with the neural specificity gene *unc-4* to govern motor neuron identity in *C. elegans***. *Development* 1997, **124**:1699-709.

5. KM Lickteig, JS Duerr, DL Frisby, DH Hall, JB Rand, DM Miller, 3rd: **Regulation of neurotransmitter vesicles by the homeodomain protein UNC- 4 and its transcriptional corepressor UNC-37/groucho in *C. elegans* cholinergic motor neurons**. *J Neurosci* 2001, **21**:2001-14.

6. BC Prasad, B Ye, R Zackhary, K Schrader, G Seydoux, RR Reed: ***unc-3*, a gene required for axonal guidance in *C. elegans*, encodes a member of the O/E family of transcription factors**. *Development* 1998, **125**:1561-1568.

7. S Garel, F Marin, MG Mattei, C Vesque, A Vincent, P Charnay: **Family of Ebf/Olf-1-related genes potentially involved in neuronal differentiation and regional specification in the central nervous system**. *Developmental Dynamics* 1997, **210**:191-205.

8. R Pocock, J Ahringer, M Mitsch, S Maxwell, A Woollard: **A regulatory network of T-box genes and the even-skipped homologue *vab-7* controls patterning and morphogenesis in *C. elegans***. *Development* 2004, **131**:2373-85.

9. X Huang, HJ Cheng, M Tessier-Lavigne, Y Jin: **MAX-1, a novel PH/MyTH4/FERM domain cytoplasmic protein implicated in netrin-mediated axon repulsion**. *Neuron* 2002, **34**:563-76.

10. DG Ahn, I Ruvinsky, AC Oates, LM Silver, RK Ho: ***tbx20*, a new vertebrate T-box gene expressed in the cranial motor neurons and developing cardiovascular structures in zebrafish**. *Mech Dev* 2000, **95**:253-8.

11. F Kraus, B Haenig, A Kispert: **Cloning and expression analysis of the mouse T-box gene *Tbx20***. *Mech Dev* 2001, **100**:87-91.

12. M Thellmann, J Hatzold, B Conradt: **The Snail-like CES-1 protein of *C. elegans* can block the expression of the BH3-only cell-death activator gene *egl-1* by antagonizing the function of bHLH proteins**. *Development* 2003, **130**:4057-71.

13. EK Nakakura, DN Watkins, KE Schuebel, V Sriuranpong, MW Borges, BD Nelkin, DW Ball: **Mammalian Scratch: a neural-specific Snail family transcriptional repressor**. *Proc Natl Acad Sci U S A* 2001, **98**:4010-5.

14. CR Gissendanner, K Crossgrove, KA Kraus, CV Maina, AE Sluder: **Expression and function of conserved nuclear receptor genes in *C. elegans***. *Dev Biol* 2004, **266**:399-416.

15. AH Ludewig, C Kober-Eisermann, C Weitzel, A Bethke, K Neubert, B Gerisch, H Hutter, A Antebi: **A novel nuclear receptor/coregulator complex controls *C. elegans* lipid metabolism, larval development, and aging**. *Genes Dev* 2004, **18**:2120-33.

16. B Appel, JS Eisen: **Retinoids run rampant: multiple roles during spinal cord and motor neuron development**. *Neuron* 2003, **40**:461-4.

17. HM Zhou, WW Walthall: **UNC-55, an orphan nuclear hormone receptor, orchestrates synaptic specificity among two classes of motor neurons in *C. elegans***. *J Neurosci* 1998, **18**:10438-44.

18. JW Much, DJ Slade, K Klampert, G Garriga, B Wightman: **The fax-1 nuclear hormone receptor regulates axon pathfinding and neurotransmitter expression**. *Development* 2000, **127**:703-12.

19. P Sengupta, HA Colbert, CI Bargmann: **The *C. elegans* gene *odr-7* encodes an olfactory-specific member of the nuclear receptor superfamily**. *Cell* 1994, **79**:971-980.

20. C Vogel, SA Teichmann, C Chothia: **The immunoglobulin superfamily in *Drosophila melanogaster* and *C. elegans* and the evolution of complexity**. *Development* 2003, **130**:6317-28.

21. H Hutter, BE Vogel, JD Plenefisch, CR Norris, RB Proenca, J Spieth, C Guo, S Mastwal, X Zhu, J Scheel, et al: **Conservation and novelty in the evolution of cell adhesion and extracellular matrix genes**. *Science* 2000, **287**:989-94.

22. G Rougon, O Hobert: **New insights into the diversity and function of neuronal immunoglobulin superfamily molecules**. *Annu Rev Neurosci* 2003, **26**:207-38.

23. B Eisenhaber, P Bork, Y Yuan, G Loffler, F Eisenhaber: **Automated annotation of GPI anchor sites: case study *C. elegans***. *Trends Biochem Sci* 2000, **25**:340-1.

24. K Shen, CI Bargmann: **The immunoglobulin superfamily protein SYG-1 determines the location of specific synapses in *C. elegans***. *Cell* 2003, **112**:619-30.

25. K Shen, RD Fetter, CI Bargmann: **Synaptic specificity is generated by the synaptic guidepost protein SYG-2 and its receptor, SYG-1**. *Cell* 2004, **116**:869-81.

26. SG Hall, AJ Bieber: **Mutations in the *Drosophila* neuroglian cell adhesion molecule affect motor neuron pathfinding and peripheral nervous system patterning**. *J Neurobiol* 1997, **32**:325-40.

27. NR Cohen, JS Taylor, LB Scott, RW Guillery, P Soriano, AJ Furley: **Errors in corticospinal axon guidance in mice lacking the neural cell adhesion molecule L1**. *Curr Biol* 1998, **8**:26-33.

28. L Chen, B Ong, V Bennett: **LAD-1, the *C. elegans* L1CAM homologue, participates in embryonic and gonadal morphogenesis and is a substrate for fibroblast growth factor receptor pathway-dependent phosphotyrosine-based signaling**. *J Cell Biol* 2001, **154**:841-55.

29. JA Zallen, SA Kirch, CI Bargmann: **Genes required for axon pathfinding and extension in the *C. elegans* nerve ring**. *Development* 1999, **126**:3679-92.

30. JA Girault, E Peles: **Development of nodes of Ranvier**. *Curr Opin Neurobiol* 2002, **12**:476-85.

31. C Faivre-Sarrailh, S Banerjee, J Li, M Hortsch, M Laval, MA Bhat: ***Drosophila* contactin, a homolog of vertebrate contactin, is required for septate junction organization and paracellular barrier function**. *Development* 2004, **131**:4931-42.

32. SJ Butler, S Ray, Y Hiromi: **klingon, a novel member of the *Drosophila* immunoglobulin superfamily, is required for the development of the R7 photoreceptor neuron**. *Development* 1997, **124**:781-92.

33. E Hill, ID Broadbent, C Chothia, J Pettitt: **Cadherin superfamily proteins in *C. elegans* and *Drosophila melanogaster***. *J Mol Biol* 2001, **305**:1011-24.

34. CH Lee, T Herman, TR Clandinin, R Lee, SL Zipursky: **N-cadherin regulates target specificity in the *Drosophila* visual system**. *Neuron* 2001, **30**:437-50.

35. M Yamagata, JR Sanes, JA Weiner: **Synaptic adhesion molecules**. *Curr Opin Cell Biol* 2003, **15**:621-32.

36. ID Broadbent, J Pettitt: **The *C. elegans* *hmr-1* gene can encode a neuronal classic cadherin involved in the regulation of axon fasciculation**. *Curr Biol* 2002, **12**:59-63.

37. G Hintsch, A Zurlinden, V Meskenaite, M Steuble, K Fink-Widmer, J Kinter, P Sonderegger: **The calsyntenins--a family of postsynaptic membrane proteins with distinct neuronal expression patterns**. *Mol Cell Neurosci* 2002, **21**:393-409.

38. GM Benian, JE Kiff, N Neckelmann, DG Moerman, RH Waterston: **Sequence of an unusually large protein implicated in regulation of myosin activity in *C. elegans***. *Nature* 1989, **342**:45-50.

39. TM Rogalski, GP Mullen, MM Gilbert, BD Williams, DG Moerman: **The UNC-112 gene in *C. elegans* encodes a novel component of cell-matrix adhesion structures required for integrin localization in the muscle cell membrane**. *J Cell Biol* 2000, **150**:253-64.

40. M Koppen, JS Simske, PA Sims, BL Firestein, DH Hall, AD Radice, C Rongo, JD Hardin: **Cooperative regulation of AJM-1 controls junctional integrity in *C. elegans* epithelia**. *Nat Cell Biol* 2001, **3**:983-91.

41. SJ Hallam, A Goncharov, J McEwen, R Baran, Y Jin: **SYD-1, a presynaptic protein with PDZ, C2 and rhoGAP-like domains, specifies axon identity in *C. elegans***. *Nat Neurosci* 2002, **5**:1137-46.

42. M Zhen, Y Jin: **The liprin protein SYD-2 regulates the differentiation of presynaptic termini in *C. elegans***. *Nature* 1999, **401**:371-5.

43. C Serra-Pages, QG Medley, M Tang, A Hart, M Streuli: **Liprins, a family of LAR transmembrane protein-tyrosine phosphatase-interacting proteins**. *J Biol Chem* 1998, **273**:15611-20.

44. EM Van Lieshout, I Van der Heijden, WJ Hendriks, CE Van der Zee: **A decrease in size and number of basal forebrain cholinergic neurons is paralleled by diminished hippocampal cholinergic innervation in mice lacking leukocyte common antigen-related protein tyrosine phosphatase activity**. *Neuroscience* 2001, **102**:833-41.

45. NX Krueger, D Van Vactor, HI Wan, WM Gelbart, CS Goodman, H Saito: **The transmembrane tyrosine phosphatase DLAR controls motor axon guidance in *Drosophila***. *Cell* 1996, **84**:611-22.

46. TR Clandinin, CH Lee, T Herman, RC Lee, AY Yang, S Ovasapyan, SL Zipursky: ***Drosophila* LAR Regulates R1-R6 and R7 Target Specificity in the Visual System**. *Neuron* 2001, **32**:237-48.

47. RJ Harrington, MJ Gutch, MO Hengartner, NK Tonks, AD Chisholm: **The *C. elegans* LAR-like receptor tyrosine phosphatase PTP-3 and the VAB-1 Eph receptor tyrosine kinase have partly redundant functions in morphogenesis**. *Development* 2002, **129**:2141-53.

48. AM Schaefer, GD Hadwiger, ML Nonet: ***rpm-1*, a conserved neuronal gene that regulates targeting and synaptogenesis in *C. elegans***. *Neuron* 2000, **26**:345-56.

49. M Zhen, X Huang, B Bamber, Y Jin: **Regulation of presynaptic terminal organization by *C. elegans* RPM-1, a putative guanine nucleotide exchanger with a RING-H2 finger domain**. *Neuron* 2000, **26**:331-43.

50. EH Liao, W Hung, B Abrams, M Zhen: **An SCF-like ubiquitin ligase complex that controls presynaptic differentiation**. *Nature* 2004.

51. JR Sanes, JW Lichtman: **Development of the vertebrate neuromuscular junction**. *Annu Rev Neurosci* 1999, **22**:389-442.

52. CW Whitfield, C Benard, T Barnes, S Hekimi, SK Kim: **Basolateral localization of the *C. elegans* epidermal growth factor receptor in epithelial cells by the PDZ protein LIN-10**. *Mol Biol Cell* 1999, **10**:2087-100.

53. C Rongo, CW Whitfield, A Rodal, SK Kim, JM Kaplan: **LIN-10 Is a Shared Component of the Polarized Protein Localization Pathways in Neurons and Epithelia**. *Cell* 1998, **94**:751-759.

54. TW Harris, K Schuske, EM Jorgensen: **Studies of synaptic vesicle endocytosis in the nematode *C. elegans***. *Traffic* 2001, **2**:597-605.

55. DH Hall, ED Hedgecock: **Kinesin-related gene *unc-104* is required for axonal transport of synaptic vesicles in *C. elegans***. *Cell* 1991, **65**:837-847.

56. DT Byrd, M Kawasaki, M Walcoff, N Hisamoto, K Matsumoto, Y Jin: **UNC-16, a JNK-signaling scaffold protein, regulates vesicle transport in *C. elegans***. *Neuron* 2001, **32**:787-800.

57. R Sakamoto, DT Byrd, HM Brown, N Hisamoto, K Matsumoto, Y Jin: **The *C. elegans* UNC-14 RUN Domain Protein Binds to the Kinesin-1 and UNC-16 Complex and Regulates Synaptic Vesicle Localization**. *Mol Biol Cell* 2005, **16**:483-96.

58. J Rand, M Nonet: **Synaptic Transmission**. In: *C. elegans II* Edited by TB D. A. Riddle, B. J. Meyer, and J. R. Priess. pp. 611-643. Cold Spring Harbor, NY: Cold Spring Harbor Press; 1997: 611-643.

59. TC Sudhof: **The synaptic vesicle cycle**. *Annu Rev Neurosci* 2004, **27**:509-47.

60. R Janz, K Hofmann, TC Sudhof: **SVOP, an evolutionarily conserved synaptic vesicle protein, suggests novel transport functions of synaptic vesicles**. *J Neurosci* 1998, **18**:9269-81.

61. R Janz, Y Goda, M Geppert, M Missler, TC Sudhof: **SV2A and SV2B function as redundant Ca2+ regulators in neurotransmitter release**. *Neuron* 1999, **24**:1003-16.

62. RM Weimer, EM Jorgensen: **Controversies in synaptic vesicle exocytosis**. *J Cell Sci* 2003, **116**:3661-6.

63. JE Richmond, KS Broadie: **The synaptic vesicle cycle: exocytosis and endocytosis in *Drosophila* and *C. elegans***. *Curr Opin Neurobiol* 2002, **12**:499-507.

64. K Iwasaki, J Staunton, O Saifee, M Nonet, JH Thomas: ***aex-3* encodes a novel regulator of presynaptic activity in *C. elegans***. *Neuron* 1997, **18**:613-22.

65. HT Kao, B Porton, S Hilfiker, G Stefani, VA Pieribone, R DeSalle, P Greengard: **Molecular evolution of the synapsin gene family**. *J Exp Zool* 1999, **285**:360-77.

66. E Takao-Rikitsu, S Mochida, E Inoue, M Deguchi-Tawarada, M Inoue, T Ohtsuka, Y Takai: **Physical and functional interaction of the active zone proteins, CAST, RIM1, and Bassoon, in neurotransmitter release**. *J Cell Biol* 2004, **164**:301-11.

67. T Shiratsuchi, K Oda, H Nishimori, M Suzuki, E Takahashi, T Tokino, Y Nakamura: **Cloning and characterization of BAP3 (BAI-associated protein 3), a C2 domain-containing protein that interacts with BAI1**. *Biochem Biophys Res Commun* 1998, **251**:158-65.

68. RE Palmer, SB Lee, JC Wong, PA Reynolds, H Zhang, V Truong, JD Oliner, WL Gerald, DA Haber: **Induction of BAIAP3 by the EWS-WT1 chimeric fusion implicates regulated exocytosis in tumorigenesis**. *Cancer Cell* 2002, **2**:497-505.

69. ML Nonet, AM Holgado, F Brewer, CJ Serpe, BA Norbeck, J Holleran, L Wei, E Hartwieg, EM Jorgensen, A Alfonso: **UNC-11, a *C. elegans* AP180 homologue, regulates the size and protein composition of synaptic vesicles**. *Mol Biol Cell* 1999, **10**:2343-60.

70. TW Harris, E Hartwieg, HR Horvitz, EM Jorgensen: **Mutations in synaptojanin disrupt synaptic vesicle recycling**. *J Cell Biol* 2000, **150**:589-600.

71. KR Schuske, JE Richmond, DS Matthies, WS Davis, S Runz, DA Rube, AM van der Bliek, EM Jorgensen: **Endophilin is required for synaptic vesicle endocytosis by localizing synaptojanin**. *Neuron* 2003, **40**:749-62.

72. Y Zhang, B Grant, D Hirsh: **RME-8, a conserved J-domain protein, is required for endocytosis in *C. elegans***. *Mol Biol Cell* 2001, **12**:2011-21.

73. AK Jones, DB Sattelle: **Functional genomics of the nicotinic acetylcholine receptor gene family of the nematode, *C. elegans***. *Bioessays* 2004, **26**:39-49.

74. S Hallam, E Singer, D Waring, Y Jin: **The *C. elegans* NeuroD homolog cnd-1 functions in multiple aspects of motor neuron fate specification**. *Development* 2000, **127**:4239-4252.

75. E Culetto, HA Baylis, JE Richmond, AK Jones, JT Fleming, MD Squire, JA Lewis, DB Sattelle: **The *C. elegans* *unc-63* gene encodes a levamisole-sensitive nicotinic acetylcholine receptor alpha subunit**. *J Biol Chem* 2004.

76. WR Schafer: **Genetic analysis of nicotinic signaling in worms and flies**. *J Neurobiol* 2002, **53**:535-41.

77. S Jones, S Sudweeks, JL Yakel: **Nicotinic receptors in the brain: correlating physiology with function**. *Trends Neurosci* 1999, **22**:555-561.

78. J Kim, DS Poole, LE Waggoner, A Kempf, DS Ramirez, PA Treschow, WR Schafer: **Genes affecting the activity of nicotinic receptors involved in *C. elegans* egg-laying behavior**. *Genetics* 2001, **157**:1599-610.

79. S Halevi, L Yassin, M Eshel, F Sala, S Sala, M Criado, M Treinin: **Conservation within the RIC-3 gene family. Effectors of mammalian nicotinic acetylcholine receptor expression**. *J Biol Chem* 2003, **278**:34411-7.

80. C Gally, S Eimer, JE Richmond, JL Bessereau: **A transmembrane protein required for acetylcholine receptor clustering in *C. elegans***. *Nature* 2004, **431**:578-82.

81. C Savage-Dunn: **Targets of TGF beta-related signaling in *C. elegans***. *Cytokine Growth Factor Rev* 2001, **12**:305-12.

82. Y Suzuki, MD Yandell, PJ Roy, S Krishna, C Savage-Dunn, RM Ross, RW Padgett, WB Wood: **A BMP homolog acts as a dose-dependent regulator of body size and male tail patterning in *C. elegans***. *Development* 1999, **126**:241-50.

83. A Colavita, S Krishna, H Zheng, RW Padgett, JG Culotti: **Pioneer axon guidance by UNC-129, a *C. elegans* TGF-beta**. *Science* 1998, **281**:706-9.

84. BD McCabe, G Marques, AP Haghighi, RD Fetter, ML Crotty, TE Haerry, CS Goodman, MB O'Connor: **The BMP homolog Gbb provides a retrograde signal that regulates synaptic growth at the *Drosophila* neuromuscular junction**. *Neuron* 2003, **39**:241-54.

85. HM Robertson: **Updating the str and srj (stl) families of chemoreceptors in Caenorhabditis nematodes reveals frequent gene movement within and between chromosomes**. *Chem Senses* 2001, **26**:151-9.

86. MK Stewart, NL Clark, G Merrihew, EM Galloway, JH Thomas: **High Genetic Diversity in the Chemoreceptor Superfamily of *C. elegans***. *Genetics* 2004.

87. ER Troemel: **Chemosensory signaling in *C. elegans***. *Bioessays* 1999, **21**:1011-20.

88. P Sengupta, JH Chou, CI Bargmann: ***odr-10* encodes a seven transmembrane domain olfactory receptor required for responses to the odorant diacetyl**. *Cell* 1996, **84**:899-909.

89. WA Catterall: **Structure and regulation of voltage-gated Ca2+ channels**. *Annu Rev Cell Dev Biol* 2000, **16**:521-55.

90. EA Mathews, E Garcia, CM Santi, GP Mullen, C Thacker, DG Moerman, TP Snutch: **Critical residues of the *C. elegans* *unc-2* voltage-gated calcium channel that affect behavioral and physiological properties**. *J Neurosci* 2003, **23**:6537-45.

91. WR Schafer, BM Sanchez, CJ Kenyon: **Genes affecting sensitivity to serotonin in *C. elegans***. *Genetics* 1996, **143**:1219-30.

92. CI Bargmann: **Neurobiology of the *C. elegans* genome**. *Science* 1998, **282**:2028-33.

93. EE Strehler, M Treiman: **Calcium pumps of plasma membrane and cell interior**. *Curr Mol Med* 2004, **4**:323-35.

94. SA Thayer, YM Usachev, WJ Pottorf: **Modulating Ca2+ clearance from neurons**. *Front Biosci* 2002, **7**:d1255-79.

95. M Ouardouz, MA Nikolaeva, E Coderre, GW Zamponi, JE McRory, BD Trapp, X Yin, W Wang, J Woulfe, PK Stys: **Depolarization-induced Ca2+ release in ischemic spinal cord white matter involves L-type Ca2+ channel activation of ryanodine receptors**. *Neuron* 2003, **40**:53-63.

96. C Harteneck, TD Plant, G Schultz: **From worm to man: three subfamilies of TRP channels**. *Trends Neurosci* 2000, **23**:159-66.

97. D Chin, AR Means: **Calmodulin: a prototypical calcium sensor**. *Trends Cell Biol* 2000, **10**:322-8.

98. A Karabinos, I Bussing, E Schulze, J Wang, K Weber, R Schnabel: **Functional analysis of the single calmodulin gene in the nematode *C. elegans* by RNA interference and 4-D microscopy**. *Eur J Cell Biol* 2003, **ban**:557-63.

99. EE Corcoran, AR Means: **Defining Ca2+/calmodulin-dependent protein kinase cascades in transcriptional regulation**. *J Biol Chem* 2001, **276**:2975-8.

100. J Bandyopadhyay, J Lee, A Bandyopadhyay: **Regulation of calcineurin, a calcium/calmodulin-dependent protein phosphatase, in *C. elegans***. *Mol Cells* 2004, **18**:10-6.

101. M Robatzek, JH Thomas: **Calcium/calmodulin-dependent protein kinase II regulates *C. elegans* locomotion in concert with a G(o)/G(q) signaling network**. *Genetics* 2000, **156**:1069-82.

102. H Hu, JH Sheehan, WJ Chazin: **The Mode of Action of Centrin: BINDING OF Ca2+ AND A PEPTIDE FRAGMENT OF Kar1p TO THE C-TERMINAL DOMAIN**. *J Biol Chem* 2004, **279**:50895-903.

103. P Ramulu, J Nathans: **Cellular and subcellular localization, N-terminal acylation, and calcium binding of *C. elegans* protein phosphatase with EF-hands**. *J Biol Chem* 2001, **276**:25127-35.

104. KR Norman, DG Moerman: **Alpha spectrin is essential for morphogenesis and body wall muscle formation in *C. elegans***. *J Cell Biol* 2002, **157**:665-77.

105. CE Creutz, SL Snyder, SN Daigle, J Redick: **Identification, localization, and functional implications of an abundant nematode annexin**. *J Cell Biol* 1996, **132**:1079-92.

106. C Miller: **An overview of the potassium channel family**. *Genome Biol* 2000, **1**:REVIEWS0004.

107. L Salkoff, A Butler, G Fawcett, M Kunkel, C McArdle, G Paz-y-Mino, M Nonet, N Walton, ZW Wang, A Yuan, et al: **Evolution tunes the excitability of individual neurons**. *Neuroscience* 2001, **103**:853-9.

108. P Phelan, TA Starich: **Innexins get into the gap**. *Bioessays* 2001, **23**:388-96.

109. T Starich, M Sheehan, J Jadrich, J Shaw: **Innexins in *C. elegans***. *Cell Commun Adhes* 2001, **8**:311-4.

110. JG White, E Southgate, JN Thomson, S Brenner: **The structure of the nervous system of the nematode *C. elegans***. *Phil. Trans. R. Soc. Lond.* 1986, **B314**:1-340.

111. L Bianchi, M Driscoll: **Protons at the gate: DEG/ENaC ion channels help us feel and remember**. *Neuron* 2002, **34**:337-40.

112. N Tavernarakis, W Shreffler, S Wang, M Driscoll: ***unc-8*, a DEG/ENaC family member, encodes a subunit of a candidate mechanically gated channel that modulates *C. elegans* locomotion**. *Neuron* 1997, **18**:107-119.

113. MB Goodman, GG Ernstrom, DS Chelur, R O'Hagan, CA Yao, M Chalfie: **MEC-2 regulates *C. elegans* DEG/ENaC channels needed for mechanosensation**. *Nature* 2002, **415**:1039-42.

114. MM Sedensky, JM Siefker, JY Koh, DM Miller, 3rd, PG Morgan: **A stomatin and a degenerin interact in lipid rafts of the nervous system of *C. elegans***. *Am J Physiol Cell Physiol* 2004, **287**:C468-74.

115. S Zhang, C Ma, M Chalfie: **Combinatorial marking of cells and organelles with reconstituted fluorescent proteins**. *Cell* 2004, **119**:137-44.

| **Cosmid Name**  **Additional File 16. Summary of *unc-4*::GFP enriched transcripts with neuronal functions.** | **Common Name** | **Rank** | **KOG (Other description)** |
| --- | --- | --- | --- |
| ***Transcription Factors***  F26C11.2  Y16B4A.1  T27A1.6  F43G9.11  W09C2.1  C49H3.5a  H01A20.1  Y39B6A.17  R11E3.5  F09C6.9  F41B5.9  C29G2.5  C27C12.6  B0336.13  F44E2.6  T08H4.3 | *unc-4*  *unc-3*  *mab-9*  *ces-1*  *elt-1*  *ntl-4*  *nhr-3*  *nhr-95*  *nhr-104*  *nhr-116* | 5  515  788  851  608  614  716  63  406  118  533  732  465  310  643  946 | Transcription factor, contains HOX domain  HLH transcription factor EBF/Olf-1 and related DNA binding proteins  TBX1 and related T-box transcription factors  C2H2-type Zn-finger protein  GATA-4/5/6 transcription factors  MOT2 transcription factor  Hormone receptors  Hormone receptors  Hormone receptors  Nuclear hormone receptor  Hormone receptors  7-transmembrane receptor  Transcription factor Doublesex  Transcription initiation factor IIA, gamma subunit  Predicted pilin-like transcription factor  Predicted transcription factor (ETS domain) |
| Adhesion Proteins  K02E10.8  C18F3.2  C33F10.5a  C53A5.13  C53B7.1  B0034.3  ZK617.1a  C47E8.7  C25A11.4a  T19D12.6 | *syg-1*  *sax-7*  *rig-3*  *cdh-11*  *unc-22*  *unc-112*  *ajm-1* | 877  381  95  305  976  859  600  895  682  784 | Immunoglobulin C-2 Type/fibronectin type III domains  (Neural Cell Adhesion Molecule L1CAM)  Neural cell adhesion molecule L1  Immunoglobulin C-2 Type/fibronectin type III domains  Immunoglobulin C-2 Type/fibronectin type III domains  Calsyntenin  Projectin/twitchin and related proteins  Mitogen inducible gene product (contains ERM and PH domains)  Unnamed protein (Apical junction molecule class)  Basement membrane-specific heparan sulfate proteoglycan (HSPG) core protein |
| Synapse Associated  F35D2.5  F59F5.6  C09D8.1  K07F5.6  C01B7.6  F07B7.12  C18H9.7  F17E5.1a  C09H6.2 | *syd-1*  *syd-2*  *ptp-3*  *rpm-1*  *rpy-1*  *lin-2*  *lin-10* | 705  348  658  906  860  423  377  484  889 | PDZ domain  LAR-interacting protein and related proteins  Protein tyrosine phosphatase  Protein tyrosine phosphatase  Inhibitor of type V adenylyl cyclases/Neuronal presynaptic protein Highwire/PAM/RPM-1  Inhibitor of type V adenylyl cyclases/Neuronal presynaptic protein Highwire/PAM/RPM-1  Acetylcholine receptor-associated protein of the synapse (rapsyn)  Calcium/calmodulin-dependent serine protein kinase/membrane-associated guanylate kinase  Beta amyloid precursor-binding protein |
| Synaptic Vesicle Trafficking  C18C4.10  B0478.1a  F35C8.3 | *klc-2*  *jnk-1*  *jkk-1* | 38  332  446 | Kinesin light chain  Jun-N-terminal kinase (JNK)  Mitogen-activated protein kinase (MAPK) kinase MKK7/JNKK2 |
| Vesicle Loading  ZC416.8b  ZC4168a  Y51A2D.18  ZK637.1 | *cha-1*  *unc-17* | 938  193  51  952 | Carnitine O-acyltransferase CRAT (Choline-acetyltransferase)  Vesicular amine transporter  Synaptic vesicle transporter SVOP and related transporters (major facilitator superfamily)  Synaptic vesicle transporter SV2 (major facilitator superfamily) |
| Exocytosis and Neurotransmitter Release  T08A9.3  Y22F5A.3  F27D9.1a  C18A3.6A  T10H9.4  F31E8.2  Y38C1BA.2  C02H7.3  F45E4.3  F54G2.1a | *sng-1*  *SNAP-25*  *unc-18*  *rab-3*  *unc-10*  *snt-1*  *snn-1*  *aex-3* | 3  544  61  27  53  845  438  262  510  548 | Synaptic vesicle protein Synaptogyrin involved in regulation of Ca2+-dependent exocytosis  SNAP-25 (synaptosome-associated protein) component of SNARE complex  Vesicle trafficking protein Sec1  GTPase Rab3, small G protein superfamily  Rab3 effector RIM1 and related proteins, contain PDZ and C2 domains  Ca2+-dependent phospholipid-binding protein Synaptotagmin, required for synaptic vesicle and secretory granule exocytosis  Synaptic vesicle protein Synapsin  MAPK-activating protein DENN  Rab3 effector RIM1 and related proteins, contain PDZ and C2 domains  Synaptic vesicle protein BAIAP3, involved in vesicle priming/regulation |
| Endocytosis  C32E8.10a  T20B5.1  F02E8.3  F29G9.3  C27H6.1  T04D1.3  F35A5.8  F18C12.2a | *unc-11*  *apt-4*  *aps-2*  *apt-2*  *apt-10*  *unc-57*  *erp-1*  *rme-8* | 263  439  764  491  662  700  561  871 | Clathrin assembly protein AP180 and related proteins, contain ENTH domain  Vesicle coat complex AP-2, alpha subunit  Clathrin adaptor complex, small subunit  Clathrin adaptor complex, small subunit  Stoned B synaptic vesicle biogenesis protein  Lysophosphatidic acid acyltransferase endophilin/SH3GL, involved in synaptic vesicle formation  SH3 domain protein SH3GLB  Endocytosis protein RME-8, contains DnaJ domain |
| Neurotransmitter-gated Metabotropic Receptors  F47D12.1  Y40H4A.1  Y41G9A.4  Y22D7AR.13  F15A8.5d | *gar-2*  *gar-3*  *ser-4*  *dop-1* | 245  421  769  680  683 | 7 transmembrane receptor (G-protein linked AChR)  Muscarinic acetylcholine receptor  GABA-B ion channel receptor subunit GABABR1 and related subunits, G-protein coupled receptor superfamily  7 transmembrane receptor (Serotonin/Octopamine Receptor family)  Predicted membrane protein (Dopamine Receptor) |
| TGF-beta Signaling  F39G3.8 | *tig-2* | 216 | Transforming growth factor beta, bone morphogenetic protein and related proteins |
| Serpentine Receptors  F34D6.5  K02E2.3  F41B5.8  T06C12.2  B0547.4  T21B4.9  R04D3.9  Y54G11A.12  Y52E8A.5  H27D07.5  K07C6.15  Y97E10B.2  C47E8.3  Y37H2C.4  F57A8.4  Y97E10B.3  C06H5.7  C17H11.1 | *sri-62*  *srh-177*  *str-253*  *str-57*  *srd-8*  *srh-70*  *srd-43*  *srh-41*  *srw-40*  *srw-122*  *srx-70* | 93  380  402  474  720  830  878  692  1004  622  820  673  369  740  745  843  958  960 | Predicted olfactory G-protein coupled receptor  Predicted olfactory G-protein coupled receptor  7-transmembrane olfactory receptor  7-transmembrane olfactory receptor  Chemoreceptor/7TM receptor  Predicted olfactory G-protein coupled receptor  Chemoreceptor/7TM receptor  Predicted olfactory G-protein coupled receptor  Uncharacterized conserved protein  7-transmembrane olfactory receptor  7-transmembrane receptor  7-transmembrane receptor  7-transmembrane receptor  7-transmembrane olfactory receptor  7-transmembrane receptor  7-transmembrane receptor  7-transmembrane receptor  7 transmembrane receptor |
| Calcium Channels  T02C5.5  T24F1.6  C11D2.6  K11C4.5  Y67D8C.10  F35C12.2  ZC21.2 | *unc-2*  *nca-1*  *unc-68*  *mca-3*  *ncx-4*  *trp-1* | 109  252  17  555  987  954  239 | Voltage-gated Ca2+ channels, alpha1 subunits  L-type voltage-dependent Ca2+ channel, alpha2/delta subunit  Voltage-gated Ca2+ channels, alpha1 subunits  Ca2+ release channel (ryanodine receptor)  Calcium transporting ATPase  K+-dependent Ca2+/Na+ exchanger NCKX1 and related proteins  Receptor-activated Ca2+-permeable cation channels (STRPC family) |
| Calcium Ion Binding  T21H3.3  Y73B3A.12  F12A10.5  F55C10.1  R08D7.5  F23H11.8  K10B3.10  ZC155.1  T08G5.10  C04B4.2 | *cmd-1*  *cnb-1*  *pef-1*  *spc-1*  *nex-1*  *mtl-2* | 16  32  654  472  454  712  214  247  929  123 | Calmodulin and related proteins (EF-Hand superfamily)  Calmodulin and related proteins (EF-Hand superfamily)  Calmodulin and related proteins (EF-Hand superfamily)  Ca2+/calmodulin-dependent protein phosphatase (calcineurin subunit B), EF-Hand superfamily protein  Ca2+-binding protein (centrin/caltractin), EF-Hand superfamily protein  Protein serine/threonine phosphatase RDGC/PPEF, contains STphosphatase and EF-hand domains  Ca2+-binding actin-bundling protein (spectrin), alpha chain (EF-Hand protein superfamily)  Annexin  Predicted metallothionein  Unnamed protein (Calcium-binding EF hand) |
| Potassium Channels  F17C8.5  R04F11.4  F46A9.3  F36A2.4  T28A8.1  Y39B6A.19  T06H11.1  R05G9.2 | *twk-6*  *twk-13*  *twk-29*  *twk-30*  *twk-40*  *twk-46*  *unc-58* | 537  437  542  329  681  6  866  90 | Tandem pore domain K+ channel  Tandem pore domain K+ channel  Tandem pore domain K+ channel  Tandem pore domain K+ channel  Tandem pore domain K+ channel  Tandem pore domain K+ channel  Tandem pore domain K+ channel  Tandem pore domain K+ channel |
| Innexins  R12H7.1 | *unc-9* | 652 | Innexin-type channels |
| DEG/EnaC  R13A1.4 | *unc-8* | 451 | Non voltage-gated ion channels (DEG/ENaC family) |
| Stomatin  K03E6.5  Y71H9A.2 | *unc-1*  *sto-6* | 461  350 | Prohibitins and stomatins of the PID superfamily  Prohibitins and stomatins of the PID superfamily |
