# Supplementary material for: A gene expression fingerprint of C. elegans embryonic motor neurons
Source: BMC Genomics. 2005 Mar 21;6:42. doi: 10.1186/1471-2164-6-42 (PMC1079822; doi:10.1186/1471-2164-6-42)
Supplement: Additional File 17 — Logic tree of data analysis methods. [file 1471-2164-6-42-S17.pdf]

Additional File 17

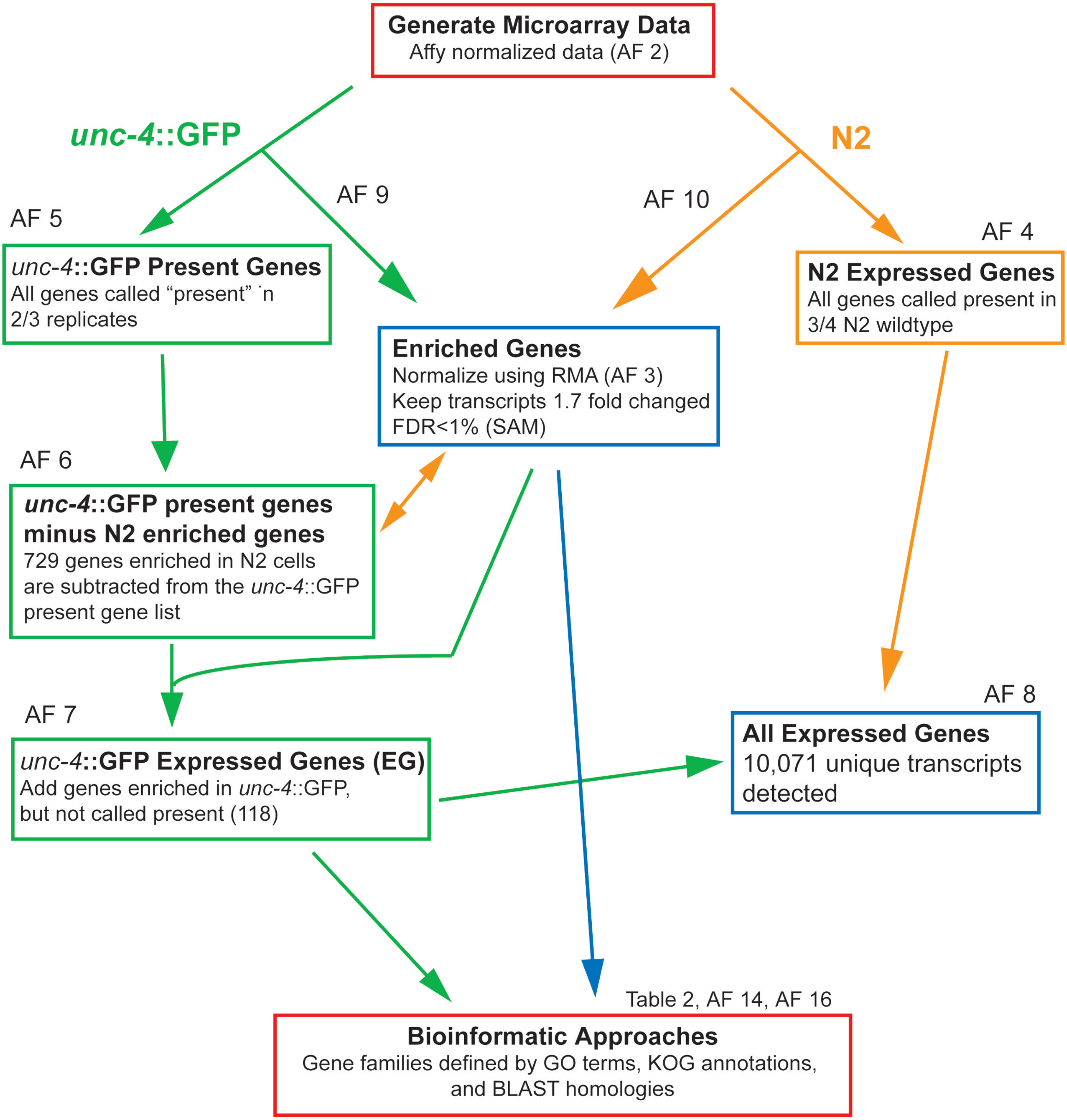

Legend - AF denotes “Additional File.” N2 refers to wildtype reference dataset derived from all embryonic cells. Orange denotes N2 dataset. Green denotes *unc-4*::GFP dataset. Blue denotes combination, while red refers to methods applied to both datasets
